# Structural basis for the increased processivity of D-family DNA polymerases in complex with PCNA

**DOI:** 10.1038/s41467-020-15392-9

**Published:** 2020-03-27

**Authors:** Clément Madru, Ghislaine Henneke, Pierre Raia, Inès Hugonneau-Beaufet, Gérard Pehau-Arnaudet, Patrick England, Erik Lindahl, Marc Delarue, Marta Carroni, Ludovic Sauguet

**Affiliations:** 1 Unit of Structural Dynamics of Macromolecules, Institut Pasteur and CNRS UMR 3528, Paris, France; 2 CNRS, Ifremer, Université de Brest, Laboratoire de Microbiologie des Environnements Extrêmes, Plouzané, France; 3 Sorbonne Université, École Doctorale Complexité du Vivant (ED515), Paris, France; 4 Utech UBI, Institut Pasteur, CNRS UMR 3528, Paris, France; 5 Molecular Biophysics Platform, C2RT, Institut Pasteur, CNRS UMR 3528, Paris, France; 6 Department of Biochemistry and Biophysics, Science for Life Laboratory, Stockholm University, Stockholm, Sweden; 7 Department of Applied Physics, KTH Royal Institute of Technology, Stockholm, Sweden

**Keywords:** Replisome, Cryoelectron microscopy

## Abstract

Replicative DNA polymerases (DNAPs) have evolved the ability to copy the genome with high processivity and fidelity. In Eukarya and Archaea, the processivity of replicative DNAPs is greatly enhanced by its binding to the proliferative cell nuclear antigen (PCNA) that encircles the DNA. We determined the cryo-EM structure of the DNA-bound PolD–PCNA complex from *Pyrococcus abyssi* at 3.77 Å. Using an integrative structural biology approach — combining cryo-EM, X-ray crystallography, protein–protein interaction measurements, and activity assays — we describe the molecular basis for the interaction and cooperativity between a replicative DNAP and PCNA. PolD recruits PCNA via a complex mechanism, which requires two different PIP-boxes. We infer that the second PIP-box, which is shared with the eukaryotic Polα replicative DNAP, plays a dual role in binding either PCNA or primase, and could be a master switch between an initiation and a processive phase during replication.

## Abstract (web summary)

Replicative DNA polymerases (DNAPs) have evolved the ability to copy the genome with high processivity and fidelity. Here, the authors present a cryo-EM structure of the DNA-bound PolD–PCNA complex from *Pyrococcus abyssi* to reveal the molecular basis for the interaction and cooperativity between a replicative DNAP and PCNA.

## Introduction

DNA replication is one of the most important functions in living organisms and viruses. It ensures the integrity of the genome and the accurate transfer of genetic information. DNA polymerases (DNAPs) are the key enzymes of DNA replication and diverse DNA repair processes^1^. Cellular organisms typically use multiple DNAPs, which have been grouped into different families based on their sequence alignments: PolA, PolB, PolC, PolD, PolX, PolY, and reverse transcriptase^2,3^. Genomic DNA replication is carried out by the so-called replicative DNAPs, which have evolved to copy the genome with high processivity and fidelity^4^. The main replicative DNAPs from Eukarya are found in family B, from Bacteria in family C, and from Archaea in families B and D. Across every domain of life, polymerase holoenzyme accessory proteins play an integral role in achieving the extraordinary efficacy and accuracy of the replicative polymerase complex. These include a sliding clamp that encircles the DNA^5^ and greatly enhances the processivity^6^. The bacterial sliding clamp is referred to as the β clamp, whereas the eukaryotic and archaeal sliding clamp protein is called the proliferative cell nuclear antigen (PCNA)^7^. Clamps are constructed from either two (β) or three monomers (PCNA) to yield a ring composed of six domains, which share similar protein folds^8,9^.

In eukaryotes, PCNA stimulates processive DNA synthesis of both lagging and leading strands upon association with DNAPs δ (Pol δ) and ε (Pol ε), respectively^10–12^. PCNA inhibition is therefore considered as a valuable anticancer strategy^13^. In Archaea, PCNA has been shown to recruit replicative DNAPs of both B- and D-families, respectively, named PolB and PolD^14,15^. Organisms within the archaeal domain of life possess a simplified version of the eukaryotic DNA replication machinery. The archaeal PCNA shares 25% identity with the human PCNA and PolD, despite having the two-barrel fold of multi-subunit RNA polymerases for its catalytic domain, shares intriguing similarities with the three main multi-subunit eukaryotic replicative DNAPs: Polα, Polδ, and Polε. In particular, the PolD DP1 subunit and the C-terminal domain of the DP2 subunit are homologous to the regulatory B-subunit and the C-terminal domain of the catalytic A-subunit, which are found in all eukaryotic replicative DNAPs^16,17^. PolD is an archaeal replicative DNAP^18,19^, which is widely distributed among Archaea (except in crenarchaea) and has been shown to be essential for cell viability^20–23^. Similar to other replicative DNAPs, the activity of PolD is strongly stimulated through its interaction with PCNA^14,15,24^. PCNA-binding partners carry short motifs known as the PCNA-interacting protein box (PIP-box), but sequence divergent motifs have been reported to bind to the same binding pocket^25^. Although the PIP-boxes are the best known PCNA-interacting peptides, other motifs including RIR and MIP motifs have been reported^26,27^. Since the first structures of sliding clamps were determined, about 100 structures have been reported, in their apo form, bound with DNA, or in complex with various PIP-boxes and other PCNA-interacting motifs^28^. However, only two structures of full-length replicative DNAPs bound with PCNA and DNA have been reported to date: the *Pyrococcus furiosus* PolB–PCNA-DNA ternary complex that was determined by negative-staining electron microscopy (EM)^29^, and the cryo-EM structure of the Human Polδ-PCNA holoenzyme, which was published while this manuscript was under revision^30^.

Here we present the cryo-EM structure of the DNA-bound PolD–PCNA complex from *Pyrococcus abyssi* at 3.77 Å using an integrative structural biology approach, combining cryo-EM, X-ray crystallography, protein–protein interaction measurements, and activity assays. This structure unveils the molecular basis for the interaction and cooperativity between the whole replicative polymerase and PCNA with an unprecedented level of detail. PolD recruits PCNA via a complex mechanism, which requires two different PIP-box motifs: a C-terminal and an internal one that has never been characterized so far. We infer that the C-terminal PIP-box, which is shared with the eukaryotic Polα replicative DNAP, plays a dual role in binding either PCNA or primase, and could be a master switch between an initiation phase and a processive phase during replication.

## Results

### Architecture of the DNA-bound PolD–PCNA processive complex

**Fig. 1:**
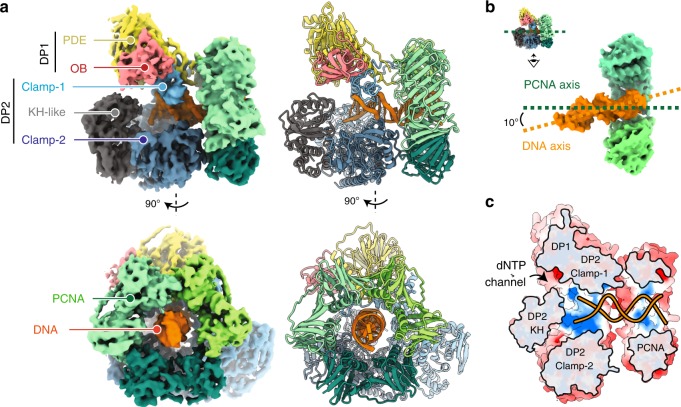
Cryo-EM structure of the DNA-bound PolD–PCNA processive complex. **a** Two orthogonal views of the cryo-EM density map (left) and cartoon representations (right) of the DNA-bound PolD–PCNA complex. **b** Orientation of the DNA duplex with respect to the PCNA threefold symmetry axis. **c** Cutaway front view of the PolD–PCNA–DNA complex showing the electrostatic surface potential with negative, neutral, and positive charges represented in red, white, and blue, respectively.

The *P. abyssi* PolD processive complex was reconstituted by incubating PCNA with the PolD exonuclease-deficient variant^31^ (DP1 H451A) in a 3 : 1 ratio, in the presence of an 18-mer primed DNA duplex with a 7-nucleotide overhang and a non-hydrolyzable nucleotide analog. The reconstituted complex (317 kDa) was vitrified and its structure was determined using single-particle cryo-EM. The map was solved at an average resolution of 3.77 Å (Table 1 and Supplementary Figs. 1 and 2). The essential PolD and PCNA DNA-binding regions, as well as the DP1–DP2 and DP2–PCNA interface regions showed a higher resolution map at 3.0–3.5 Å (Fig. 1a and Supplementary Fig. 2). In these regions, the density map of the DNA-bound PolD–PCNA complex was of sufficient quality to allow de novo building of the majority of the protein. The map includes several regions for which no atomic model was known before, such as regions neighboring the active site and the DP1–DP2 interface. In the peripheral region of the complex, the DP2 KH domain, the DP1 OB domain, and some regions of the PCNA were found to be flexible and the local resolution map ranged between 4.0 and 6.0 Å (Supplementary Fig. 2). In these regions, crystal structures of PolD DP1 (144–619) and DP2 (1–1050) individual subunits^17^, and the structure of the *P. abyssi* PCNA (from this study using X-ray crystallography at 2.3 Å resolution) were used in model building. DNA was docked into the cryo-EM map, guided by the density for the duplex region showing minor and major grooves, as well as the unambiguous position of purines and pyrimidines (Supplementary Fig. 3). However, no obvious density for single-stranded DNA and the incoming nucleotide was observed in the DP2 active site.

**Table 1:** Cryo-EM data collection, refinement, and validation statistics.

	PolD–PCNA–DNA complex (EMDB-10401) (PDB 6TH8)
Data collection and processing	
Magnification	165,000
Voltage (kV)	300
Electron exposure (e–/Å^2^)	40
Defocus range (μm)	−0.5 to −3.5
Pixel size (Å)	0.83
Symmetry imposed	C1
Initial particle images (no.)	269,251
Final particle images (no.)	147,511
Map resolution (Å)	3.77
FSC threshold	0.143
Map resolution range (Å)	3 to 6
Refinement	
Initial model used (PDB code)	5IJL, 5IHE, 6T7X
Model resolution (Å)	3.77
FSC threshold	0.143
Model resolution range (Å)	3 to 6
Map sharpening *B* factor (Å^2^)	145.192
Model composition	
Non-hydrogen atoms	19,398
Protein residues	2347
Ligands	5
*B* factors (Å^2^)	
Protein	81.31
Ligand	131.54
R.m.s. deviations	
Bond lengths (Å)	0.012
Bond angles (°)	1.258
Validation	
MolProbity score	2.58
Clashscore	38.66
Poor rotamers (%)	0.94
Ramachandran plot	
Favored (%)	91.21
Allowed (%)	8.66
Disallowed (%)	0.13

A defining feature of the PolD–PCNA–DNA ternary complex is its compactness (Supplementary Movie 1): the radius of the PCNA ring and the clamp-like PolD DNA-binding domain match perfectly (Fig. 1a). The structure of PCNA in the complex is not distorted compared with the structure of free PCNA and we conclude that the cryo-EM structure represents a stable interaction of DNA-bound PolD with a closed PCNA clamp. Away from the active site, PCNA surrounds one helix turn of the nascent DNA duplex, which is located at the center of the PCNA ring. The nascent DNA duplex runs straight through PCNA and adopts an almost perpendicular orientation (∼80°) with respect to the DNA (Fig. 1b). Interaction with PCNA nearly doubles the positively charged surface formed by the PolD active site, making a 60 Å-long DNA-binding site (Fig. 1c). This structure thus rationalizes how PCNA enhances the processivity of PolD: interaction with PCNA perpetuates the interactions with the nascent DNA duplex when it exits the PolD clamp, thereby preventing the polymerase from falling off prematurely.

### PolD–DNA complex

**Fig. 2:**
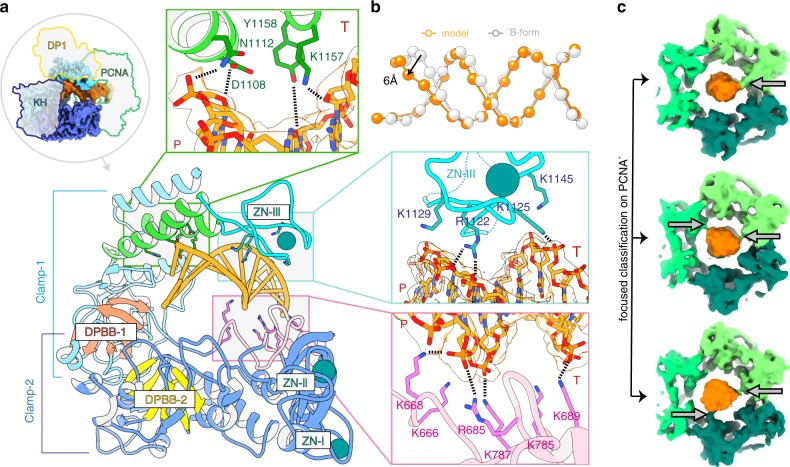
Structural basis for DNA binding by the PolD–PCNA complex. **a** View of the DP2 clamp-1 (light blue) and clamp-2 (dark blue) domains surrounding one turn of the DNA duplex (orange). The two catalytic double-psi β-barrels DPBB-1 and DPBB-2 are represented in red and yellow, respectively. Template and primer strands are indicated by T and P, respectively. **b** Interaction with the PolD clamp causes a local distortion of the DNA (orange) compared with an ideal B-form DNA (white). The phosphates of the DNA duplexes are shown as spheres. **c** PCNA makes labile contacts with DNA. Focused classification on PCNA resulted in three 3D classes showing extra-densities between DNA and PCNA residues K84 and K86, which are underlined by arrows.

The primer-template is held in position in the PolD active site by a bipartite clamp domain. Clamp-1 and clamp-2 domains contribute a central cleft located upstream from the DP2 catalytic center, bordered with positively charged side chains, which encircles one helix turn of the nascent DNA (Fig. 2a). Although the DNA bound by the PolD–PCNA complex is predominantly in the B-form, interaction with the PolD clamp causes a distortion of the DNA region located next to the active site. Indeed, five base pairs at the primer 3′-end are distorted, showing a decreased helical twist and a widened minor groove (Fig. 2b). The clamp-1 domain contains a Zn-binding module, named Zn-III, connected to two α-helices that pushes against the minor groove of DNA. The Zn-III module harbors four conserved basic residues that interact intimately with the phosphodiester backbone: R1122 and K1129 interact with the primer strand whereas K1125 and K1145 contact the template strand. On the opposite side, the clamp-2 domain binds to the minor groove of the DNA, with numerous interactions between the side chains of five canonical lysines (K666, K668, K689, K785, and K787) and both the primer and the template strands. A similar widening of the minor groove has been observed in the DNA-bound structures of other DNAPs associated with proofreading activities. In A-, B-, and C-family DNAPs, conserved tyrosine, arginine, or lysine residues have been shown to interact with the minor groove and to participate in the catalytic efficiency^32–34^. Minor groove hydrogen-bonding interactions between DNAPs and N3 of purines or O2 of pyrimidines contribute to the efficiency of DNA synthesis and base selectivity. Similarly, the structure of PolD shows two canonical residues K1157 and Y1158 pointing towards universal hydrogen bond acceptors at purines N3 and pyrimidines O2 positions, which may be important for the catalytic efficiency and fidelity of PolD.

### PCNA–DNA binding interactions

Interestingly, PCNA and PolD adopt strikingly different DNA-binding modes. Although PolD extensively interacts with the nascent DNA duplex, the PCNA channel exposes several positively charged residues, which are pointed towards the DNA backbone and make labile transient polar contacts with DNA. Consistently, unlike the DNA region present in the PolD active site, the structure of DNA does not appear to be influenced by its interaction with PCNA and adopts a perfect B-form DNA architecture (Fig. 2b). The local resolution of the outer perimeter of PCNA is about 4.0–6.0 Å, substantially lower than the average resolution of the consensus map (3.77 Å), indicating a greater flexibility in the PCNA–DNA interactions compared with the PolD–DNA interactions. To characterize further the molecular determinants of the PCNA sliding movement, we performed a focused three-dimensional (3D) classification on PCNA and identified three classes showing extra densities between DNA and the PCNA residues K84 and K86 (Fig. 2c). Interestingly, DNA was found to be in contact with different monomers of PCNA in the three 3D classes, suggesting that all three PCNA monomers contribute to DNA binding through short-lived polar contacts. Such labile transient interactions between DNA and PCNA have already been observed in another study using an integrative structural biology approach combining nuclear magnetic resonance and molecular dynamics simulations^35^. Thus, PCNA provides an electrostatic cushion for the DNA to pass through as it leaves the PolD active site, thereby allowing it to rapidly slide onto DNA, pulled by PolD.

### PolD–PCNA interface

**Fig. 3:**
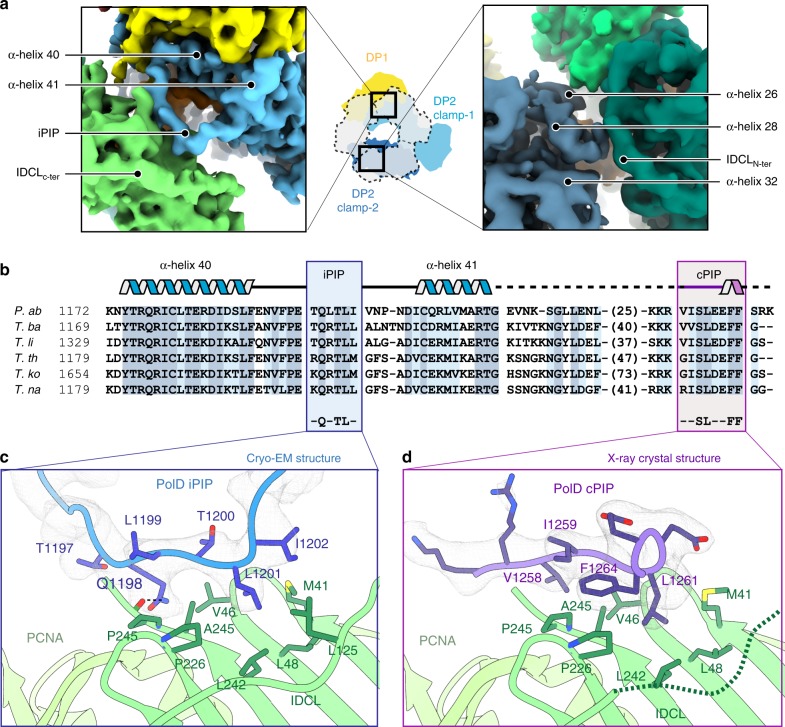
PolD uses two distinct PIP-boxes for molecular recognition of PCNA. **a** Two views of the cryo-EM map showing the interfacial region between the DP2 clamp-1 (left) and clamp-2 (right) binding to PCNA. DP2 clamp-1 and clamp-2 domains are represented in light and dark blue, respectively; DP1 is shown in yellow; DNA in orange; PCNA in green. **b** Multiple-sequence alignment of the C-terminal region in Thermococcus species: *P. abyssi* (P.ab), *Thermococcus barophilus* (T.ba), *Thermococcus litoralis* (T.li), *Thermococcus thioreducens* (T.re), *Thermococcus kodakarencis* (T.ko), and *Thermococcus nautili* (T.na). Internal (iPIP) and C-terminal (cPIP) PIP-boxes are framed in blue and purple, respectively, with secondary structure elements shown above. Sequence similarities are highlighted with light blue boxes and conserved residues are highlighted with dark blue boxes. **c** Detailed view of the iPIP–PCNA interaction in the cryo-EM map contoured at a level of 7*σ*. **d** Detailed view of the cPIP–PCNA interaction in the 2Fo-Fc X-ray electron density map contoured at a level of 1.5*σ*.

The cryo-EM map shows how PCNA is tethered to PolD through multiple contacts that involve both clamp-1 and clamp-2 domains of DP2 (Fig. 3a). First, the α-helices, α-40, and α-41 of the clamp-1 domain are connected by a loop of 17 amino acids, which is hooked into PCNA. This loop binds to the canonical PCNA PIP-binding pocket through an internal PIP-box (iPIP), which has never been identified so far (Fig. 3b). Six residues within this iPIP fill the PCNA PIP-binding pocket. Among them, the side chain of Q1198 penetrates deep into the pocket, making contacts with the canonical PCNA residue P245 (Fig. 3c). In addition, the bulky side chains of L1199, L1201, and I1202 make extensive contacts with hydrophobic residues, which line the PCNA PIP-binding pocket. Interestingly, the PolD iPIP shows a non-canonical structure compared with other PIP-boxes, lacking a 4-residue 3_10_-helix turn, which has been observed in the structures of all PIP–PCNA complexes determined so far^28^.

Second, the N-terminal region of the interdomain-connecting loop of the adjacent PCNA monomer binds to the DP2 clamp-2 domain (Fig. 3a). This interaction is mediated through polar contacts between residues E692, K779, Y781, and K896 from clamp-2 and PCNA residues H75, D117, and E119 (Supplementary Fig. 4). This PCNA–clamp-2 interaction, which includes a large proportion of polar contacts, contrasts with the PCNA–iPIP site-specific interaction described above. The PCNA–clamp-2 interaction may thus be easily broken or profoundly remodeled, enabling the PCNA ring to form a new interface with PolD, when the polymerase encounters a damage and adopts an editing mode. Such a conformational transition has been proposed for the PolB–PCNA complex, when PolB switches from the polymerase state to the proofreading state^29,36^.

### PolD uses two distinct PIP-boxes for recognition of PCNA

In addition to the iPIP that was identified from our cryo-EM structure, the DP2 subunits of PolD from *P. furiosus* and *P. abyssi* have been shown to host a C-terminal PIP-box^15,24^. This second PIP-box (hereafter referred to as canonical PIP-box (cPIP)), is connected to DP2 by a 40-residue linker, which is variable in both length and amino-acid composition across archaea (Fig. 3b). Strikingly, both cPIP and the linker are not visible in the cryo-EM density of the DNA-bound PolD–PCNA complex. To better characterize the role of cPIP, we co-crystallized PCNA from *P. abyssi* with a 12 amino-acid peptide mimicking the DP2 cPIP and solved its structure at 2.7 Å resolution (Supplementary Table 1 and Supplementary Fig. 5). The final model includes 9 of the 12 amino acids of the co-crystallized peptide. In contrast to the structure of iPIP, which differs from other structures of PIP-boxes, cPIP shares the same overall fold (Fig. 3d) as those described in the literature^28^. Hence, the cPIP structure shows an extended peptide chain, whose C-terminal region folds into a 3_10_ helix. Several conserved hydrophobic residues of the cPIP—V1258, I1259, L1261, and F1264—insert their bulky side chains into the hydrophobic cleft formed by the PIP-binding pocket on the PCNA surface. It is noteworthy that cPIP lacks the consensus Q residue, which is present in most PIP-boxes^28^. The cryo-EM and crystal structures reveal that cPIP and iPIP adopt redundant binding positions in the PCNA PIP-binding pocket. Indeed, the side chains of L1199 and L1201 in iPIP and the side chains of I1259 and L1261 in cPIP are accommodated similarly in the PCNA-binding pocket, suggesting that the binding of iPIP and cPIP to PCNA are mutually exclusive. One may ask whether cPIP could not bind to one of the neighboring PCNA subunits, but no extra density that could be accounted for by cPIP was found in the cryo-EM map. Furthermore, the linker region connecting cPIP to DP2 can hardly cover the 70 Å distance, which separates the last defined residue of DP2 from the nearest unoccupied PIP-binding pocket. Altogether, these observations strongly suggest that iPIP and cPIP interact with PCNA through different mechanisms.

**Fig. 4:**
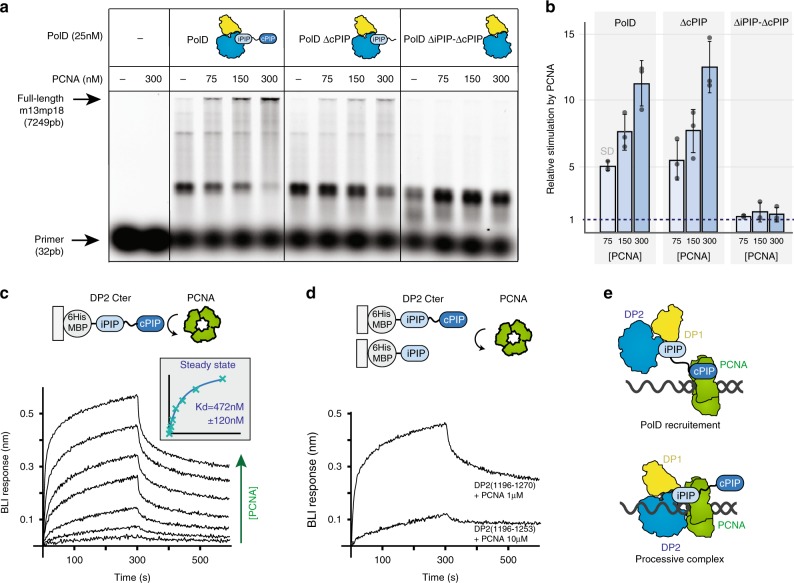
The iPIP and cPIP PIP-boxes interact with PCNA through different mechanisms. **a** Primer extension studies were performed using M13mp18 template (7 nM), hybridized to a fluorescent-labeled primer. Reactions contained 25 nM of DNAP, in the presence or absence of PCNA (75, 150, and 300 nM). **b** Quantification of the stimulation by PCNA of PolD wild type, PolDΔcPIP, and PolDΔiPIP-ΔcPIP. Histograms show full-length 7249 bp (%) for extension reaction in the presence of PCNA relative to full-length 7249 bp (%) without PCNA for each PolD. PolD wild type (*n* = 3), PolDΔcPIP (*n* = 3), and PolDΔiPIP-ΔcPIP (*n* = 3). Error bars represent 1 SD. **c** Specific binding of immobilized-DP2(1196–1270) to PCNA measured by biolayer interferometry (BLI). Steady-state analysis was performed using the average signal measured at the end of the association step (between 290 and 300 s). **d** Comparative binding of immobilized-DP2(1196–1270) and -DP2(1196–1253) to PCNA measured by BLI. The range of concentrations used in the binding experiments are listed in the Methods section. **e** Hypothetical two-steps mechanism for PCNA recruitment by PolD. Source data are provided as a Source Data file.

To assess the role of these two PIP-boxes, primer extension reactions were carried out in the presence of a primer-template of large size and increasing amounts of PCNA, to stimulate full-length DNA synthesis (Fig. 4a, b). Using primed-M13mp18 DNA template, PolD progressively became stimulated upon increasing the concentration of PCNA. At 300 nM PCNA, the maximum amount of full-length DNA products was reached. However, full-length DNA synthesis by PolDΔiPIP-ΔcPIP, devoid of both C-terminal PIP-boxes, was never obtained upon increasing PCNA concentrations even at 300 nM PCNA. We verified that the abolishment of the functional interaction of PolDΔiPIP-ΔcPIP with PCNA was not due to an intrinsically catalytic-incompetent PolDΔiPIP-ΔcPIP, as full-length DNA products were detectable over a higher range of PolDΔiPIP-ΔcPIP mutant concentrations (Supplementary Fig. 6). In contrast, PolDΔcPIP, devoid of the C-terminal cPIP only, displayed functional interaction with PCNA, yielding similar amounts of full-length DNA products to PolD wild type (Fig. 4a, b). Albeit the C-terminal cPIP is important for the physical interaction with PCNA (see below) (Fig. 4c, d), these results show that the cPIP is dispensable for full-length DNA synthesis by the PolD–PCNA complex in vitro. Altogether, these results show that iPIP, but not cPIP, is required for full-length DNA synthesis by the PolD–PCNA complex. Consistently, although the iPIP was found to bind the PCNA PIP-binding pocket, the cPIP was not visible in the cryo-EM density of the PolD–PCNA–DNA ternary complex. Together, these results suggest that PolD may be recruited by PCNA through a two-step mechanism (Fig. 4e). First, PCNA is recruited by PolD through its interaction with the DP2 cPIP. Once the PolD–PCNA complex is loaded on DNA, the complex is stabilized by an interaction between PCNA and iPIP, as observed in the cryo-EM structure, whereas cPIP becomes dispensable.

### cPIP is a dual PCNA/primase-binding peptide shared with Polα

**Fig. 5:**
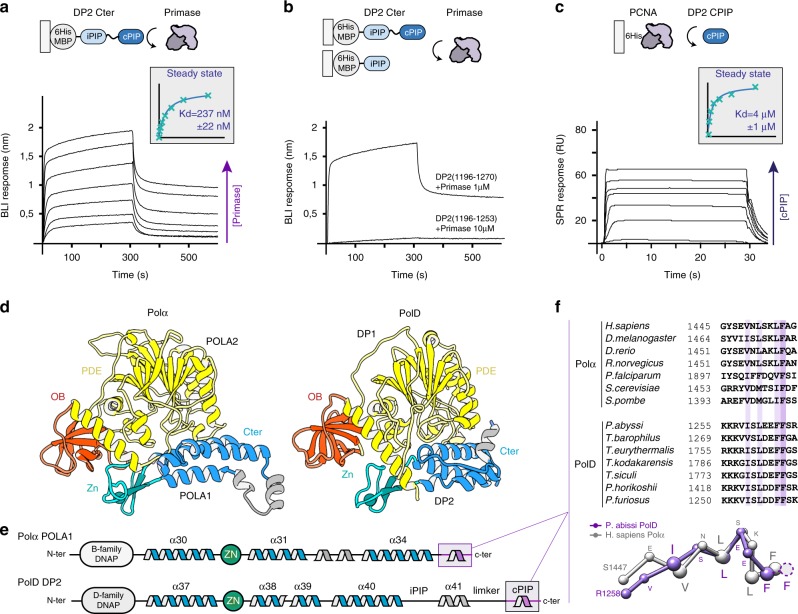
Shared primase-binding peptide in archaeal PolD and eukaryotic Polα. **a** Specific binding of immobilized-DP2(1196–1270) to primase measured by biolayer interferometry (BLI). Steady-state analysis was performed using the average signal measured at the end of the association step (between 290 and 300 s). **b** Comparative binding of immobilized-DP2(1196–1270) and -DP2(1196–1253) to primase measured by BLI. **c** Specific binding of immobilized primase with increasing concentrations of cPIP by surface plasmon resonance (RU: resonance units). Steady-state analysis was performed using the average signal measured at the end of the association step. The range of concentrations used in the binding experiments are listed in the Methods section. **d** Structural comparison of the *P. abyssi* PolD DP1-DP2(1093–1216) region of the cryo-EM structure with the *Homo sapiens* Polα POLA2-POLA1(1319–1456) crystal structure (PDB ID: 5EXR). **e** Shared structural features between archaeal PolD and eukaryotic Polα C-terminal regions. Conserved α-helices and Zn-binding domain are shown in blue and green, respectively. **f** The cPIP of DP2 resembles the primase-interacting motif located in the C terminus of Polα. Top panel: multiple-sequence alignment highlighting the conservation between the PolD cPIP motifs from *Thermococcus* species and the primase-interacting peptides of Polα. Sequence similarities are highlighted with light purple boxes, whereas conserved residues are shown with dark purple boxes. Bottom panel: superimposition of the X-ray crystal structures of the cPIP from *P. abyssi* and the primase-interacting peptide of *H. sapiens* Polα (PDB ID: 5EXR). Cα-traces are represented as ball and sticks. Conserved residues are highlighted using larger spheres. Source data are provided as a Source Data file.

PolD shares, with its eukaryotic counterparts, unifying features of their subunit organization that reveal a clear evolutionary relationship. The eukaryotic replicative DNAPs Polα, Polδ, and Polε possess a catalytic subunit, often referred to as the A-subunit, constitutively associated with different cohorts of regulatory proteins among which B-subunits are present in all three DNAP assemblies^32^. Both OB and PDE domains of DP1 share a remarkable degree of 3D structural similarity with the regulatory B-subunits of all eukaryotic replicative DNAPs^17,37^. In addition, the C-terminal region of their catalytic subunits, which is dedicated to interaction with their B-subunits, resembles the C-terminal region of DP2 that is required for interaction with DP1 (ref.^16^). In addition to their structural similarities (Fig. 5d), PolD shares common functional features with Polα, which is tightly associated with the DNA primase in a complex called primosome that is required for initiating DNA replication in eukaryotic cells^38^. Similarly, PolD has been shown to interact with the DNA primase^39^ and is able to extend RNA primers^14^, suggesting that PolD is required for initiating DNA replication in archaea. Previously, a short conserved motif located at the extreme C terminus of Polα was shown to be critical for the interaction with the primase^40–42^. We tested whether the C-terminal region of PolD, which is homologous to that of Polα (Fig. 5e), could host a similar primase-interacting peptide.

To assess the role of the C terminus of PolD in the interactions with PCNA and primase, we performed biolayer interferometry (BLI) experiments using His_6_-tagged maltose-binding protein (MBP) fusions of the C-terminal region of DP2, which were captured via surface-linked Ni-NTA. As expected, the MBP–iPIP-cPIP fusion (DP2:1196–1270) was found to readily bind to PCNA, with a *K*_D_ of 472 ± 120 nM (Fig. 4c). Interestingly, the same construct was also able to interact with primase, with a measured *K*_D_ of 237 ± 22 nM (Fig. 5a), which is very similar to the *K*_D_ of 245 nM that was reported for the interaction within the C terminus of yeast Polα and the primase^42^. Deleting cPIP in the MBP–iPIP-ΔcPIP fusion (DP2:1196–1253) strongly impaired binding to PCNA and abrogated binding to the DNA primase (Figs. 4d and 5b), showing that cPIP is a dual PCNA/primase-binding peptide. Using His_6_-tagged PCNA and His_6_-tagged primase captured via surface-linked Ni-NTA, we then performed surface plasmon experiments, allowing us to measure the ability of PCNA and primase to bind a synthetic peptide encompassing the cPIP. The cPIP binds to PCNA with a lower affinity (*K*_D_ of 49 ± 4 μM), which differs by two orders of magnitude from the one observed for the MBP–iPIP–cPIP fusion (Supplementary Fig. 7), suggesting that the flanking region may be important for binding to PCNA. Consistently, the affinity of the PIP-box for PCNA can be modulated over four orders of magnitude by positive charges in the flanking regions^28^. Interestingly, cPIP binds to the primase substantially better than to PCNA, with a *K*_D_ of 4.0 ± 1 μM (Fig. 5c). The ability of cPIP to recruit both PCNA and primase is consistent with the dual role of PolD in DNA replication initiation and elongation, which requires interaction with both partners.

Interestingly, comparing the structures of the cPIP of PolD and the primase-binding motif of Polα reveals that both peptides fold in a one-turn 3_10_ helix (Fig. 5f). This structural similarity is underpinned by the conservation of four hydrophobic and aromatic residues. The side chains of these four conserved hydrophobic residues become buried at the hydrophobic protein-peptide interface in both PolD–PCNA (Fig. 3d) and Polα-primase^40,41^. Moreover, hotspot residues that were shown to be important for primase binding^42^ such as L1451 and F1455, are particularly well-conserved in archaea (Fig. 5f). In archaea and eukaryotes, the primase forms a heterodimer composed of a small PriS subunit with the polymerase activity and a larger regulatory PriL subunit. The Polα primase-binding peptide binds onto a hydrophobic edge of the PriL subunit^40,41^. Interestingly, in archaeal PriL, the exposed hydrophobic surface is buried by a short α-helix, which fills the space occupied by the DNAP in the Polα–primase complex^41^. This suggests that either cPIP binds to another site in the archaeal primase or that the corresponding region is remodeled upon cPIP binding. Such differences in the mode of interaction between the primase and these two polymerases may be accounted for by the fact that although eukaryotic Polα forms a stable and constitutive complex with primase, PolD and primase form only a transient complex.

## Discussion

In contrast with cellular transcriptases and ribosomes, which evolved by accretion of complexity from a conserved catalytic core, it is striking that DNA replication was reinvented several times during evolution and that no replicative DNAP family is universally conserved. Bacteria, Archaea, and Eukarya have evolved three distinct protein folds to replicate their genomes as follows: (i) the Polβ-like fold—found in bacterial Pol-III, (ii) the Klenow-like fold—found both in archaeal/eukaryotic B-family DNAPs and in bacterial Pol-I, and (iii) the two-barrel fold—the third structural class of DNAPs that was recently unveiled with the structure of archaeal PolD^17,43^. Although structurally divergent, all replicative DNAPs share unifying features. Hence, across every domain of life, the extraordinary efficacy of the replicative polymerase complex is dependent on their interaction with sliding clamps, which encircle DNA and greatly enhance their processivity^7^.

The structure of the DNA-bound PolD–PCNA complex from *P. abyssi* unveils the molecular basis for the interaction and cooperativity between the PolD replicative DNAP and PCNA. Away from the PolD active site, PCNA surrounds one helix turn of the nascent DNA duplex, perpetuating the interactions with the nascent DNA duplex, thereby preventing the polymerase from falling-off prematurely. Although PolD makes extensive and strong contacts with the DNA minor groove, PCNA contributes to DNA binding through short-lived polar contacts, which provides an electrostatic cushion for the DNA to pass through as it leaves the PolD active site, thereby allowing PCNA to rapidly slide onto DNA, pulled by PolD. Despite belonging to structurally distinct classes of DNAPs, the archaeal PolD–PCNA complex (a two-barrel fold DNAP), the bacterial PolIII–clamp–exonuclease-τ_c_ complex (a Polβ-like fold DNAP)^44^, and the eukaryotic Polδ-PCNA holoenzyme (a Klenow-like fold DNAP)^30^ share intriguing structural features. In all three structures, the nascent DNA duplex runs straight through PCNA and adopts an almost perpendicular orientation with respect to the DNA. This view contrasts with the highly tilted double-stranded DNA in the crystal structure of the β-clamp DNA complex (∼22°)^45^ and in the PCNA–DNA complex (∼40°)^35,46^, suggesting that DNA binding by the clamp is versatile and strongly influenced by the polymerase.

PIP-boxes often exist in multiple copies in DNAPs. Polη and Polδ have three PIP-boxes, which contribute differentially to distinct biological functions^47,48^. We have shown that PolD uses two distinct PIP-boxes for molecular recognition of PCNA, which are located in the C-terminal region of their DP2 subunit. Strikingly, these two PIP-boxes contribute differentially to PCNA recruitment. Based on an integrative structural biology approach, combining cryo-EM, X-ray crystallography, protein–protein interaction measurements, and activity assays, we hypothesize that PolD may be recruited by PCNA through a two-step mechanism (Fig. 3e). First, PCNA is recruited by PolD through its interaction with the DP2 cPIP. Once the PolD–PCNA complex is loaded on DNA, the complex is stabilized by an interaction between PCNA and iPIP, as observed in the cryo-EM structure, whereas cPIP becomes dispensable. This mechanism is supported by a former study on PolD from *P. abyssi*, showing that removing the cPIP did not disrupt the physical interaction with PCNA, when both partners are bound to DNA^24^.

In addition, we have shown that cPIP is not only important for recruiting PCNA but does also interact with the DNA primase, a key actor of the replisome. The interplay at the replisome in hyperthermophilic archaea is of special interest as their DNA is exposed to elevated temperatures (up to 113 °C), which promote increased level of DNA damage^49^. It is striking that these archaeal species manage to maintain their genome, with a reduced repertoire of DNAPs. Although human cells are known to contain at least 17 different DNAPs^50^, the hyperthermophilic archaeon *P. abyssi* only possesses three distinct DNAPs: PolD, PolB, and the DNA primase polymerase. Recent gene deletion studies on hyperthermophylic Euryarchaea have demonstrated that only PolD is required for viability, suggesting that PolD is solely responsible for DNA replication, whereas PolB may be required for DNA repair^21,22,51^. It is noteworthy that the situation is different in Crenarchaea, which do not possess PolD^52^. Due to the multiple biological reactions required during DNA replication, PolD must be able to switch from one replication factor to another in a spatially and temporally regulated process. Indeed, our work shows that cPIP has overlapping specificities and is capable of binding both PCNA and primase. Hence, PolD must be able to interact with the primase during the initiation of DNA replication and with PCNA to ensure processive extension of both leading and lagging strands. The versatility of cPIP may be instrumental in such process. This finding expands current views on PCNA interactions showing that PIP-boxes are a much broader class of motifs than initially thought, which form the network of interacting proteins responsible for DNA replication and repair^25^.

**Fig. 6:**
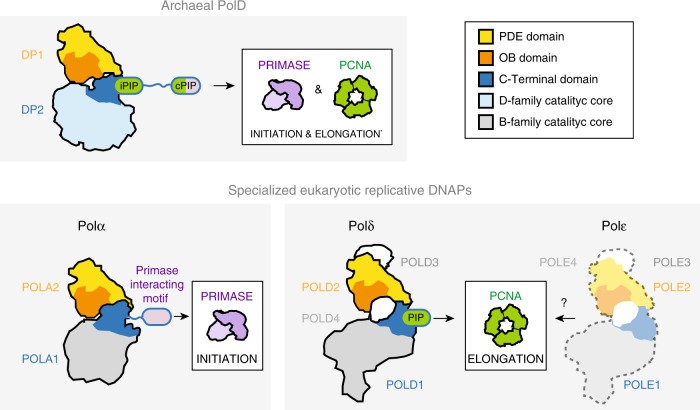
Comparison of multi-subunit polymerases in Archaea and Eukarya. The binding modes of PCNA and primase are illustrated in a schematic way and the two PIP motifs (cPIP and iPIP). The schematic representations are derived from the structures of the human Polα-primase complex (PDB ID: 5EXR), the human Polδ-PCNA complex (PDB ID: 6TNY), and the *P. abyssi* PolD–PCNA complex (this study). No structures are currently available for the PCNA–Polε complex, but its schematic representation is supported by the literature^68^.

In eukaryotes, chromosomal replication is accomplished primarily by three distinct DNAPs, which play different roles in DNA replication: Polα, Polδ, and Polε^32^. Polα is tightly associated with the primase in a constitutive complex, named the primosome, which is responsible for initiating DNA replication^38^. Polδ and Polε have been shown, in a series of experiments, to be responsible for lagging and leading strand replication, respectively^12,53^. Although they have diverged to acquire specific biological activities, all these three polymerases share unifying structural features that they most probably inherited from a common ancestor with PolD (Fig. 6)^16,54^. Both OB and PDE domains of DP1 share a remarkable degree of 3D structural similarity with the regulatory B-subunits of all eukaryotic replicative DNAPs^17,37^. In addition, the C-terminal region of their catalytic subunits, which is dedicated to interaction with their B-subunits resembles the C-terminal region of DP2 that is required for interaction with DP1 (refs^16,55^). Using an integrative structural biology approach, we identify here a conserved primase-interacting peptide conserved in PolD and Polα. This finding extends the structural similarities between the archaeal and eukaryotic multi-subunit replicative DNAPs, suggesting that their common ancestor was associated with the primase. However, the C-terminal PIP-boxes of PolD are not conserved in Polδ and Polε. We hypothesize that eukaryotic DNAPs evolved distinct mechanisms for recruiting PCNA, when the two-barrel D-family catalytic core found in PolD was exchanged by a Klenow-like B-family catalytic core, which is found in all contemporary eukaryotic replicative DNAPs. Altogether, elucidating the structure of the PolD–PCNA DNA-bound complex clarifies the evolutionary relationships with its eukaryotic counterparts and sheds light on the domain acquisition and exchange mechanism that occurred during the evolution, from the simpler replisome that prevailed in the last common eukaryotic-archaeal ancestor, to the more complex eukaryotic one.

## Methods

### Cloning, protein expression, and purification

PolD from *P. abyssi* was coexpressed by 1 mM isopropyl β-d-1-thiogalactopyranoside (IPTG) induction in *Escherichia coli* strains BL21 (DE3) grown overnight in Lysogeny broth (LB) at 20 °C and copurified by Ni-NTA and heparin chromatography (GE Healthcare), followed by tobacco etch virus (TEV) cleavage and size-exclusion chromatography. The purified PolD was concentrated to 2 mg/ml in 20 mM Tris HCl (pH 8), 200 mM NaCl, 3 mM MgCl_2_ storage buffer^16^. The DNA coding sequence of DP2(1196–1253) and DP2(1196–1270) were inserted in a pIVEX His-MBP-TEV plasmid allowing the expression of a TEV-cleavable N-terminal His_6_-MBP tag. The open reading frame (ORF) of the PCNA gene from *P. abyssi* was optimized and synthesized by GeneArt (Thermo Fisher) and inserted into pet28-a(+) plasmid with a Thrombine-cleavable N-terminal 6 × His tag. The ORFs of the PriS and PriL(1–210) genes from *P. abyssi* were also optimized and synthesized commercially by GeneArt (Thermo Fisher) and inserted into pRSFduet(+) as a polycistronic construct with a TEV-cleavable N-terminal His_14_-tagged PriS fusion protein. Production and purification of PolD constructions, PCNA, and PriS-PriL(1–210) were performed as follows: proteins were expressed in BL21-CodonPlus (DE3)-RIPL strain from *E. coli* (Agilent) at 37 °C in LB medium supplemented with 100 μg mL^−1^ of antibiotic (kanamycin or ampicillin, depending on the plasmid) and 25 μg mL^−1^ chloramphenicol. Recombinant protein expression was induced by adding 1 mM IPTG. Cells were then incubated overnight at 20 °C, collected by centrifugation, resuspended in buffer A (50 mM Na-HEPES at pH 8, 500 mM NaCl, 20 mM imidazole) supplemented with complete EDTA-free protease inhibitors (Roche), and lysed with a Cell-Disruptor. Lysates were then heated for 10 min at 60 °C (except for the two MBP-fused DP2 constructs) and loaded onto 5 mL HisTrap columns (GE Healthcare) connected to an ÄKTA purifier (GE Healthcare). Elution was performed using a linear gradient of imidazole (buffer B, 50 mM Na-HEPES at pH 8, 500 mM NaCl, 0.5 M imidazole). The protein fractions were then combined, dialyzed in buffer C (20 mM Na-HEPES pH 8, 0.1 M NaCl), loaded onto 5-ml Heparin HiTrap HP columns (GE Healthcare) and eluted with a linear gradient, by mixing buffer C with buffer D (20 mM Na-HEPES pH 8, 1 M NaCl). Purifications were finally polished using exclusion-size chromatography in buffer E (20 mM Na-Hepes pH 8, 0,15 M NaCl) on a Superdex 75 10/300 or Superdex 200 10/300 (GE Healthcare) depending of the purified protein molecular weight.

### Sample preparation for Cryo-EM

The DNA duplex was prepared by mixing equivalent molar amounts of primer (5′-CGCCGGGCCGAGCCGTGC-3′) and template (5′-AGGTCGTGCACGGCTCGGCCCGGCG-3′). The mix was then heated 2 min at 90 °C and slowly cooled to room temperature. PolD–PCNA–DNA complexes were associated by mixing 0.4 µM PolD and 1.2 µM PCNA with 0.7 µM DNA in the presence of 0.1 mM dAMPCPP in buffer F (20 mM Na-Hepes pH 8, 0.01 mM NaCl, 2 mM Mg-Acetate). The mixture was incubated for 20 min at 4 °C and pipetted onto glow-discharged holey carbon cryo-EM grids (C-flat 2/2, 4Cu, 50). Grids were frozen in liquid ethane by using a Vitrobot Mark IV (ThermoFischer) at 100% humidity, 22 °C temperature, blotting force 20, and blotting time of 4 s.

### Cryo-EM data acquisition and image processing

Movies were collected using EPU software on a Titan Krios electron microscope (Thermo Fisher Scientific) operated at 300 kV, on a GatanK2 Summit direct electron detector coupled with a Bioquantum energy filter with 20 eV slit. The defocus range was between −0.5 and −3.5 µm, the pixel size was 0.83 Å/pixel and the total dose was ∼40 electrons/Å^2^, distributed into 40 frames. Image pre-processing until two-dimensional (2D) classification was performed during data acquisition using the Scipion package^56^. Images were imported and movie frames were aligned using MotionCor2 (ref.^57^) with dose compensation applied. The contrast transfer function (CTF) was estimated with Gctf^58^ and particles were automatically picked using the Xmipp supervised picker after training was provided to tell particle from no-particles over ~1000 manually picked molecules^59^. Particles were extracted 2× binned into a 150 × 150 pixel box to perform 2D classification for cleaning on-the-fly on consecutive batches of 20,000 particles each. Around 700,000 particles were automatically picked from 4602 micrographs and after 2D classification around 270,000 particles were kept, after excluding those belonging to poorly-resolved 2D classes.

Three initial models were generated by ab initio reconstruction using stochastic gradient descent in Relion 3.0 (ref.^60^). At this point, good particles were again extracted without binning on 300 × 300 pixel box size and 3D refinement was run using one of the initial models. After 3D refinement, a 3D classification with 3 classes and local search was performed, to separate possible conformations. The classification resulted in two classes displaying sharp details and a third class with less-defined features. No obvious conformational differences were noticed and the 3D classes better resolved were combined and 3D refined to a final consensus map at 3.77 Å (gold standard 0.143 Fourier shell correlation (FSC) criterion) resolution from around 150,000 particles. CTF-refinement and Bayesian polishing were also carried on, but they did not improve the overall resolution nor the quality of the map.

To separate different conformational states, focused 3D classification upon signal subtraction was performed using masks to focus on the PCNA part of the replicative complex. 3D classification without alignment led to 5 classes allowing the identification of 3 different types of weak contacts between PCNA and DNA. Classification was done in Relion using both a *T* parameter of 4 or 100 with the intent to catch some more details at higher *T*-value. The results were comparable.

A summary of the full workflow is provided in Supplementary Figs. S1 and S2.

### Building and refinement of cryo-EM model

The density map of the DNA-bound PolD–PCNA complex was of sufficient quality to allow de novo building of the majority of the protein in COOT^61^. In the peripheral region of the complex, the DP2 KH domain, the DP1 OB domain and some regions of the PCNA were found to be more flexible and the local resolution map ranged between 4.0–4.5 Å. In these regions, model building was guided by the crystal structures of PolD DP1 (144–619) and DP2 (1–1050) individual subunits^17^, and the structure of the *P. abyssi* PCNA, which was solved in this study using X-ray crystallography at 2.3 Å resolution (PDB IDs: 5IJL, 5IHE and 6T7X). Concerning the DNA building, an ideal B-form DNA duplex was docked in the density as a starting point. The initial model was then subjected to global real-space refinement program from the PHENIX suite^62^ using secondary structure restraints. The refined model was further manually inspected and adjusted in COOT. The final model was validated with statistics from Ramachandran plots and MolProbity scores^63^. All figures were prepared using UCSF Chimera^64^ and UCSF Chimera X^65^.

### Crystallization, X-ray data collection, and processing

PCNA crystallization trials were performed at 18 °C using the hanging drop vapor diffusion technique in 2 µL drops (1 : 1 reservoir to protein ratio) equilibrated against 500 µL of reservoir solution. For the PCNA–cPIP complex, PCNA was pre-incubated 30 min with a twofold molar excess of cPIP (KKRVISLEEFFS) (Smart Bioscience) in buffer E prior crystallization trials. PCNA crystals were obtained in 20% PEG 400, 0.2 M CaCl_2_, and 0.1 M MES pH 5.5 with a PCNA solution at 5 mg mL^−1^, whereas PCNA–cPIP complex crystals were obtained in 30% PEG 400, 0.2 M MgCl_2_, and 0.1 M Bis-Tris pH 7.1 with a PCNA–cPIP complex solution at 10 mg mL^−1^. The crystals were cryoprotected by soaking in a 1 : 1 paraffin : paratone oil mix.

X-ray data were collected at the European Synchrotron Radiation Facility on beamlines ID23 and ID29 and at the SOLEIL synchrotron on beamlines PX1 and PX2. Data sets were indexed using XDS, scaled and merged with Aimless (from the CCP4 program suite (Collaborative Computational Project 1994)^66^, and corrected for anisotropy with the STARANISO server (staraniso.globalphasing.org). PCNA X-ray structure was solved by molecular replacement using the structure of PCNA from *P. furious* (PDB ID: 5AUJ). Molecular replacements were carried out with the Phaser program from Phenix^62^ and subsequent rebuilding and refinement were achieved with COOT^61^ and BUSTER^67^. Coordinates and structure factors of the PCNA and cPIP-bound PCNA structures from *P. abyssi* were deposited in the Protein Data Bank under accession codes 6T7X and 6T7Y, respectively.

### BLI assays

BLI experiments were performed on an Octet RED384 instrument (ForteBio). His-MBP-fused DP2 constructs were captured at a 1.5 nm density on Ni-NTA biosensors. Binding to PCNA and PriS-PriL(1–210) proteins was monitored for 300 s at 25 °C in buffer E supplemented with 0.2 mg mL^−1^ bovine serum albumin. Seven proteins concentrations were assayed (31.25, 62.5, 125, 250, 500, 1000, and 2000 nM) and a buffer-only reference was subtracted from all curves. Affinities were determined by fitting the concentration dependence of the experimental steady-state signals, using the Octet RED data analysis v11 software (ForteBio).

### Surface plasmon resonance assays

Surface plasmon resonance experiments were performed using a Biacore T200 instrument (GE Healthcare). All measurements were performed at 25 °C in buffer E supplemented with 100 µM EDTA. A series S sensor chip NTA (GE Healthcare) was used to immobilize ~2000 RU of His-tagged PCNA and PriS-PriL(1–210) on two of the four flowcells, and His-tagged MBP on a third as a reference. Ten concentrations (0, 0.75, 1.5, 3, 6, 12, 25, 50, 100, and 200 µM) of cPIP-peptide (KKRVISLEEFFS) (Smart Bioscience) were injected for 30 s at 30 µL min^−1^ over the three flowcells. The raw sensograms were processed by subtracting both the signals measured on the reference flowcell and the signals measured for blank injections. Corrected data were analyzed with the BIA evaluation software (GE Healthcare), by fitting the concentration dependence of the experimental steady-state signals.

### Primer extension assays

Extension reactions of the fluorescent-labeled 32-mer primer 5′-Cy5-TGCCAAGCTTGCATGCCTGCAGGTCGACTCTA-3′ annealed to the single-stranded circular M13mp18 template (7 nM) were performed in 12.5 µl of 50 mM Tris (pH 8.0), 1 mM dithiothreitol, 50 mM NaCl, 5 mM MgCl_2_, and 200 µM each of dNTPs in the presence or absence of PCNA at the indicated concentrations. DNA polymerization was initiated by addition of DNAPs (25 nM) and was conducted at 60 °C for 30 min. Reactions were quenched on ice by addition of one volume of 90% deionized formamide and 20 mM EDTA, before heating at 95 °C for 5 min. Products were resolved on 1% (w/v) alkaline agarose gel. DNA markers (2-Log DNA ladders, New England Biolabs) were loading in 45% deionized formamide and 10 mM EDTA, and run into the same gel at 4 °C for 14 h 30 min at 30 V. After electrophoresis, gels were stained with SYBR Gold (Invitrogen). Gels were first scanned with a Mode Imager Typhoon 9500 (GE Healthcare) for Cy5 to visualize the Cy5-labeled products and then scanned for SYBR Gold for detecting the ladders. Full-length 7249 bp (%) corresponds to the intensity of 7249 bp bands as a percentage of total lane intensity. In all cases, the background value was subtracted. The mean of percentage ± SD of full-length 7249 bp (%) from three independent experiments were obtained (the raw data are provided in Supplementary Table 2).

### Reporting summary

Further information on research design is available in the Nature Research Reporting Summary linked to this article.

## Supplementary information

Supplementary Information

Peer Review

Reporting Summary

Description of Additional Supplementary Files

Supplementary Movie 1

## Data Availability

Coordinates and structure factors for the PCNA and the PCNA–cPIP complex were deposited in the Protein Data Bank under the accession codes PDB 6T7X and PDB 6T7Y, respectively. The cryo-EM map of the PolD–PCNA–DNA ternary complex is deposited in the Electron Microscopy Data Bank under accession code EMD-10401. The atomic coordinate of the complex are deposited in the Protein Data Bank under accession code PDB 6T8H. The source data underlying Figs. 4c, d and 5a–c, and Supplementary Figs. 6 and 7, and Supplementary Table 2 are provided as a Source Data file. Other data are available from the corresponding authors.

## Source data

Source data file
