# Supplementary material for: Structural basis for the increased processivity of D-family DNA polymerases in complex with PCNA
Source: Nat Commun. 2020 Mar 27;11:1591. doi: 10.1038/s41467-020-15392-9 (PMC7101311; doi:10.1038/s41467-020-15392-9)
Supplement: Supplementary file 1 — Supplementary Information [file 41467_2020_15392_MOESM1_ESM.pdf]

Structural basis for the increased processivity of D-family DNA polymerases in complex with PCNA

Madru et al.

Supplementary Information

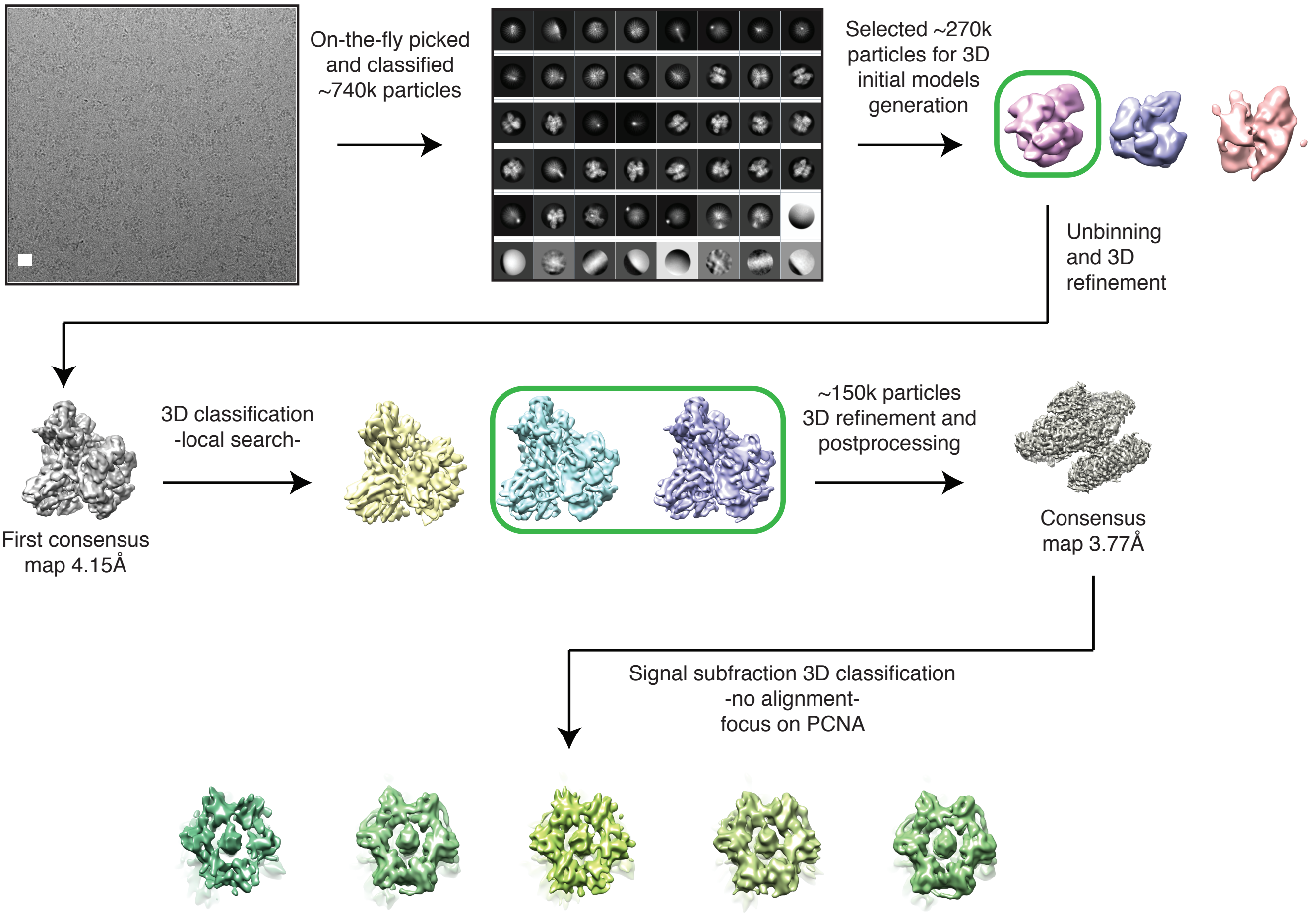

Supplementary Figure 1: Flowchart of cryo-EM data processing

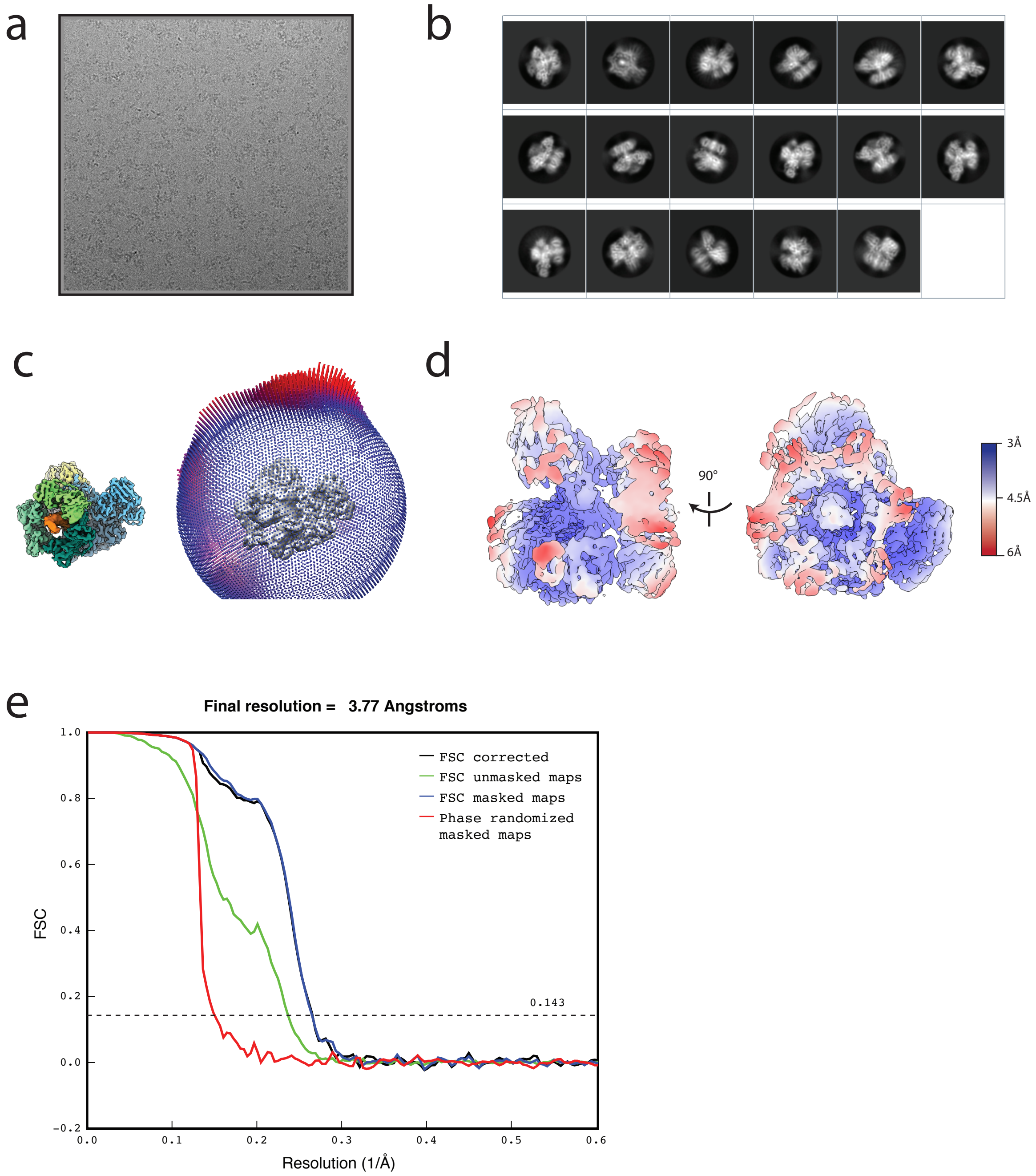

Supplementary Figure 2: Cryo-EM structure analysis of the PolD-PCNA-DNA complex. **(a)** Representative cryo-EM micrograph. **(b)** 2D class averages of the cryo-EM particles. **(c)** Angular distributions of cryo-EM particles in the final round of refinement. **(d)** Two orthogonal views of the local resolution map. **(e)** Gold-standard Fourier shell correlation (FSC) curves, showing the overall nominal resolutions of the cryo-EM maps.

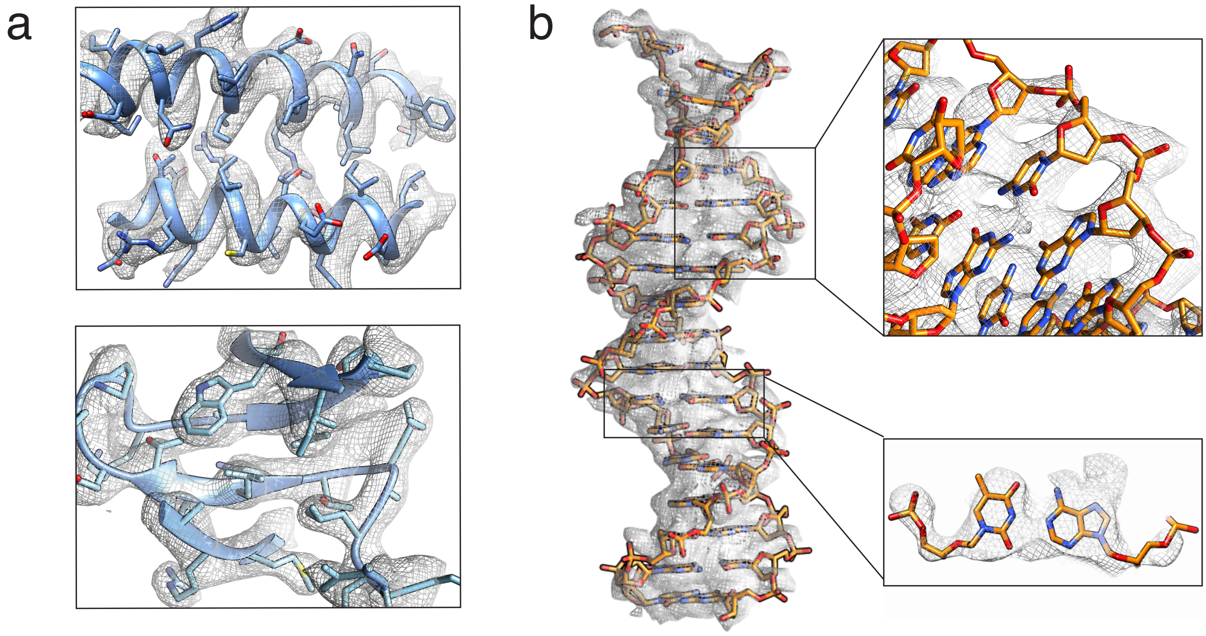

Supplementary Figure 3: Cryo-EM reconstruction of the PolD-PCNA-DNA complex. (a) Representative views of the cryo-EM map showing two α-helices (on top) and a β-sheet (on bottom) of DP2. **(b)** Representative view of the dsDNA built in density. The two additional panels illustrate the quality of the map allowing us to discriminate purines and pyrimidines.

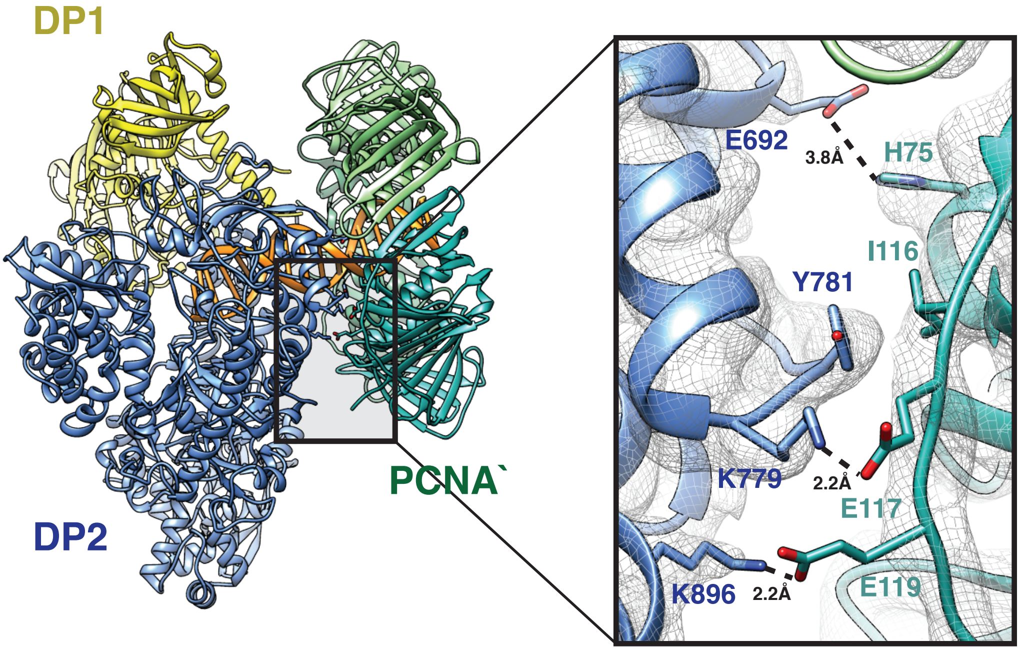

Supplementary Figure 4: Enlarged view of the contacts between DP2 clamp-2 domain and PCNA. The cryo-EM map is shown as a mesh at a level of 7 σ.

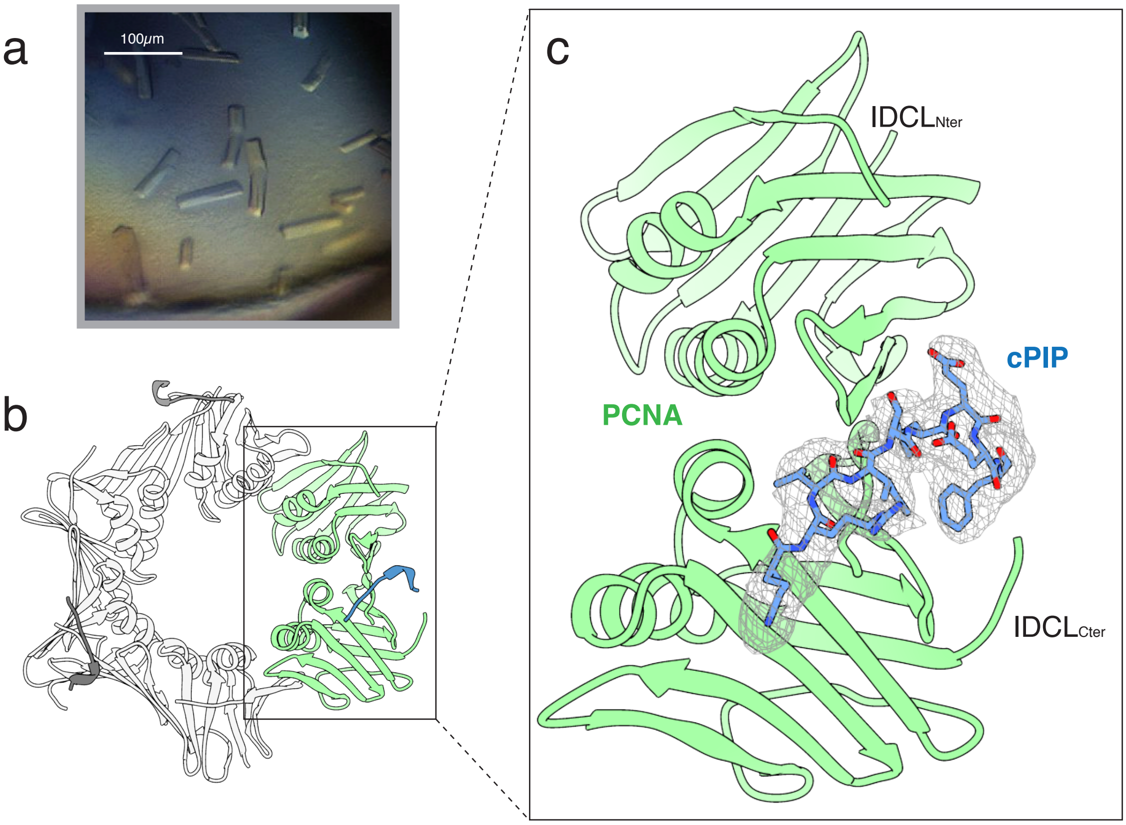

Supplementary Figure 5: Crystal structure of the cPIP-bound PCNA from P. abissy. (a) Crystals of the PCNA-cPIP complex. (b) Ribbon representation of the PCNA-cPIP complex with two crystallographic symmetry-related copies shown in grey, reconstituting the PCNA trimer. (c) Detailed view of the PCNA-cPIP complex. The X-ray 2Fo-Fc electronic-density surrounding the cPIP is represented as a mesh, contoured at a level of 1.5 σ.

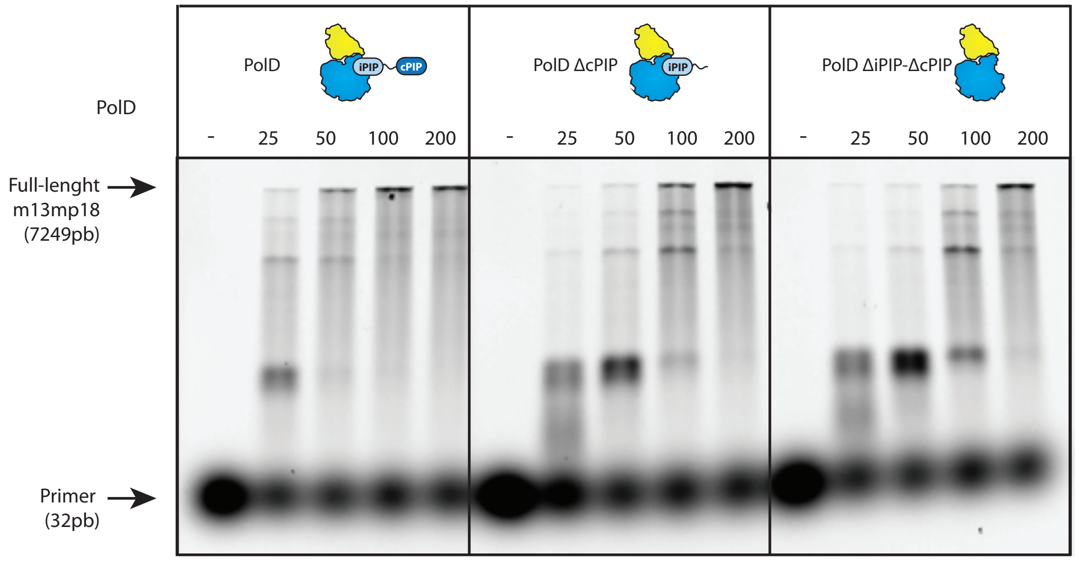

Supplementary Figure 6: PIP-boxes deletions do not affect the ability of PolD to synthetize full length DNA fragments at higher ranges of polymerase concentration. Primer extension studies were performed using M13mp18 template (7nM), hybridized to a fluorescent-labelled primer. Reactions were carried out in the presence of increasing concentration of PolD (See methods). Source data are provided as a Source Data file.

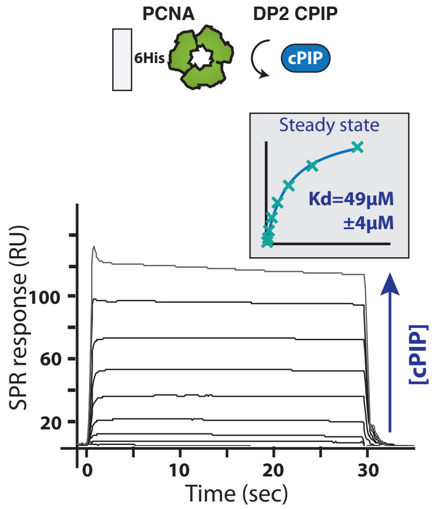

Supplementary Figure 7: Interaction between immobilized-PCNA and a synthetic cPIP peptide. Specific binding of immobilized-PCNA with increasing concentrations of cPIP by Surface Plasmon Resonance (RU: resonance units). Steady-state analysis was performed using the average signal measured at the end of the association step. The range of concentrations used is listed in the method section. Source data are provided as a Source Data file.

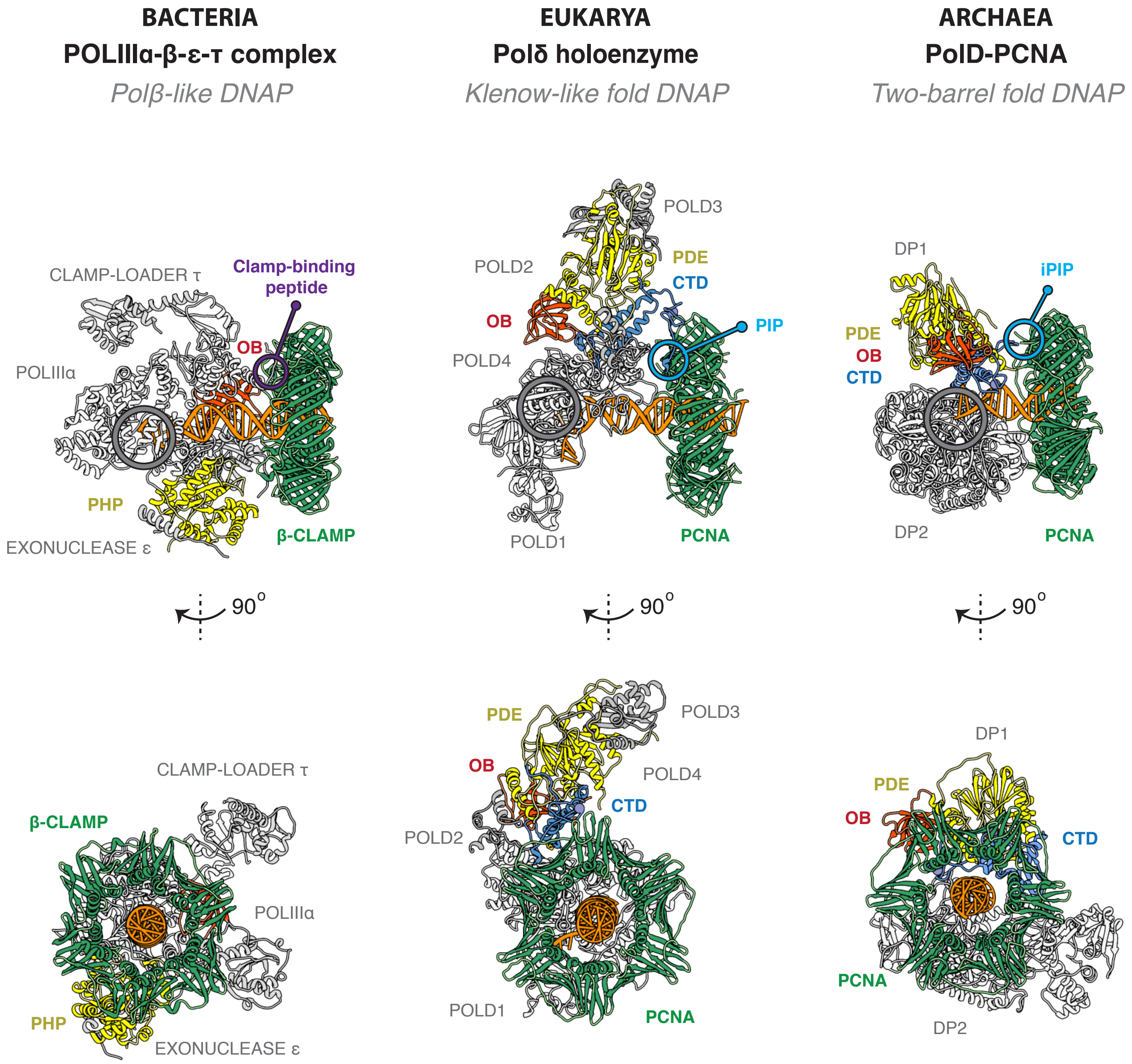

Supplementary Figure 8: Structural comparison of DNAP/sliding-clamp complexes in all three domains of life. Cartoon representations of the E. coli POLIII-clamp-exonuclease- τ500 complex (PDB ID: 5FKV), the H. sapiens Pol∂ holoenzyme (PDB ID: 6TNY) and the P. abyssi PolD-PCNA complex cryo-EM structures. Sliding-clamp interacting motifs are surrounded by purple and blue circles, whereas DNAP catalytic cores are encircled in grey.

	PCNA (PDB ID: 6T7X)	PCNA-cPIP (PDB ID: 6T7Y)
**DATA COLLECTION**		
Space group	P63	R3
a, b, c (Å)	91.90, 91.90, 64.19	140.73, 140.73, 38.52
α, β, γ (˚)	90, 90, 120	90, 90, 120
Wavelength (Å)	0.9786	0.9786
Resolution (Å)	20-2.30 (2.48-2.30)	40-2.71 (2.80 - 2.71)
R_{merge}	0.27 (1.8)	0.05 (3.94)
I/σI	14.02 (0.42)	14.79 (0.35)
$cc_{1/2}$ / cc_*	0.99 (0.32) / 1 (0.70)	0.999 (0.17) / 1 (0.54)
Completeness (%)	99.78 (99.86)	89.4 (23.48)
Redundancy	14 (10.9)	5.2 (5.1)
**REFINEMENT**		
Total reflections	193671 (15071)	40009 (3977)
Unique reflections	13821 (1387)	7716 (185)
R_{work} / R_{free}	0.202 (0.30) / 0.23 (0.31)	0.186 (0.35) / 0.262 (0.42)
N**b.** of atoms **(**Protein**)**	1877	1966
Water	56	14
Average B-factor ($Å_2$)	73.23	134.05
**VALIDATION**		
RMSD bond lengths (Å)	0.01	0.02
RMSD bond angles (˚)	1.24	2.13
Ramachandran favored	227 (97.01)	223 (92.92%)
Ramachandran allowed	7 (2.99)	17 (7.08%)
Ramachandran outliers	0 (0%)	0 (0%)

Supplementary Table 1: X-ray diffraction data collection and refinement statistics

		Full-length m13mp18 (%)			Full-length m13mp18 with PCNA (%) / Full-length m13mp18 without PCNA (%)			
		ASSAY 1	ASSAY 2	ASSAY 3	ASSAY 1	ASSAY 2	ASSAY 3	AVERAGE
**PolD WT**	PCNA 0nM	1.20	0.62	0.90	-	-	-	-
	PCNA 75nM	5.83	2.98	4.90	4.86	4.81	5.44	5.04 ± 0.35
	PCNA 150nM	8.70	4.02	8.17	7.25	6.48	9.08	7.60 ± 1.33
	PCNA 300nM	14.62	5.75	11.08	12.18	9.27	12.31	11.26 ± 1.72
**ΔcPIP**	PCNA 0nM	0.40	0.11	0.42	-	-	-	-
	PCNA 75nM	2.09	0.78	1.72	5.23	7.09	4.10	5.47 ± 1.51
	PCNA 150nM	3.25	1.00	2.47	8.13	9.09	5.88	7.70 ± 1.65
	PCNA 300nM	4.47	1.62	4.86	11.18	14.73	11.57	12.49 ± 1.95
**ΔiPIP ΔcPIP**	PCNA 0nM	0.30	0.10	0.35	-	-	-	-
	PCNA 75nM	0.40	0.12	0.41	1.33	1.20	1.17	1.23 ± 0.09
	PCNA 150nM	0.32	0.24	0.40	1.07	2.40	1.14	1.54 ± 0.75
	PCNA 300nM	0.34	0.20	0.34	1.13	2.00	0.97	1.37 ± 0.55

Supplementary Table 2: Primer extension assays raw data. Full-length m13mp18 (%) corresponds to the intensity of 7249bp bands as a percentage of total lane intensity.
