# Supplementary material for: Structural basis for the increased processivity of D-family DNA polymerases in complex with PCNA
Source: Nat Commun. 2020 Mar 27;11:1591. doi: 10.1038/s41467-020-15392-9 (PMC7101311; doi:10.1038/s41467-020-15392-9)
Supplement: Supplementary file 4 — Description of Additional Supplementary Files [file 41467_2020_15392_MOESM4_ESM.docx]

**Description of Additional Supplementary Files**

File name: Supplementary Movie 1

Description: Cryo-EM structure of the PolDPCNA holoenzyme
