# Supplementary material for: Structural basis for the increased processivity of D-family DNA polymerases in complex with PCNA
Source: Nat Commun. 2020 Mar 27;11:1591. doi: 10.1038/s41467-020-15392-9 (PMC7101311; doi:10.1038/s41467-020-15392-9)
Supplement: Supplementary file 2 — Peer Review [file 41467_2020_15392_MOESM2_ESM.pdf]

Reviewers' comments:

Reviewer #1 (Remarks to the Author):

Madru and colleagues use an integrated approach that combines protein-protein interaction studies, X-ray crystallography and cryo-EM to describe the structure of the archaeal PolD-PCNA-DNA holoenzyme. They find that PolD contacts DNA via two distinct PIP motifs. Intriguingly, one of the PIP boxes is shared with the eukaryotic DNA polymerase Pol alpha.

This is a very thorough study. In particular the cryo-EM structure reported (at resolution better than 3.8 Å) is a tremendous achievement. This study should definitely be published in Nature Communications, after minor revisions. I summarise below a few points that the authors should consider when preparing the revised version of their manuscript.

Major point:

1. Although the figures are very nicely prepared and clear, it would be extremely useful to include a movie separately depicting the structure of Pol D, the interaction between polymerase and DNA, polymerase and PCNA and PCNA and DNA.

Minor points:

1. One key reference is incorrectly cited "March, M.D. et al." should be "De March, M. et al."
2. Related to the point above, a more thorough comparison should be drawn between the interaction mode observed between PCNA and DNA in the cryo-EM map of the archaeal holo-complex, and in the crystal structure of the human PCNA-DNA crystal structure. In particular, three distinct interaction modes between PCNA-DNA are resolved with focused classification and presented in Figure 2C. Are these interaction modes compatible with the cogwheel model proposed by De March et al ?
3. In the introduction the authors state "In eukaryotes, PCNA helps to recruit replicative DNAPs δ (Pol δ) and ε (Pol ε)". While this statement is correct for Pol δ, I am not sure that this is accurate for ε. In fact ε is recruited onto the CMG helicase before the initiation step of DNA replication. I suggest focusing on the observation that PCNA stimulates processive DNA synthesis of both leading and lagging strands.
4. The statement "The local resolution in the cryo-EM map surrounding the PCNA is about 4-4.5 Å" is unclear. I would say the outer perimeter of PCNA is 4-4.5 Å. Actually, would it not be better to claim 4-6 Å ? (please note that my remark does not affect any of the conclusions of this study).
5. The sentence "The large proportion of polar contacts underlines the plasticity of the latter interaction, which contrasts with the site-specific PCNAclamp-1 interaction." is difficult to read and could be revised.
6. "suggesting that PolD is required for initiating DNA replication in Archaea". Archaea should be lower case.
7. Page 15. "The defocus range was between −0.5 and −3.5". This value does not match that report in Table S1.
8. Figure S2 has a few problems. Panel C, it would be good to overlay angular distribution and cryo-EM maps. Panel D – arrow indicating rotation should be inverted!! Panel E – should the threshold not be 0.143?
9. Table S1. Please revise "B -actors (Å2)". Also consistency in measured defocus range (see methods, page 15). The same applies to map resolution range. Is it 3 to 4.5A as stated in ? 3 to 5A as stated in table S1? Or 3 to6 A as shown by the color code?

Reviewer #2 (Remarks to the Author):

In this manuscript, Madru et al describe the cryo-EM structure of a PolD-PCNA-DNA complex from P. abyssi. In addition to reporting various aspects of the structure – many of which confirm previously reported interactions between individual components of the complex – the authors conduct a biochemical analysis of interactions made by the two PIP boxes on PolD. These results suggest that, while only one of the two PIP boxes can apparently interact with PCNA at a time, both are required for optimal PCNA binding. Additionally, the authors demonstrate that the C-terminal PIP (cPIP) is also capable of binding to primase via motifs similar to those found in eukaryotic polymerases.

Overall, I think that the combined structural and biochemical data provide nice insights into how archaeal PolD interacts with PCNA, and how this interaction may also allow primase to be transiently recruited. The study will be of interest to those working on the structure and evolution of DNA polymerases, and to the archael community. However, in light of a recent structure of human Pol ∂ in complex with PCNA and Fen1 (Lancey et al., https://doi.org/10.1101/872879), I am not convinced that this work will reach a broad audience. I have a couple of suggestions that I think would improve the manuscript, but ultimately think this manuscript is better suited to a more specialized journal.

Major critique
I think that functional assays that extend beyond protein-protein interaction Kds would add a lot to this work. For example, how do cPIP and iPIP mutations affect priming, primer extension activity, and processivity of the PolD- PCNA complex on DNA at relatively physiological concentrations of protein and NTPs/dNTPs? Given the fairly high Kds of some of the interactions (e.g. 4 micromolar for the cPIP-Primase interaction), it would be interesting to determine whether these interactions are essential or more stimulatory.

Minor critique
It would be helpful to summarize the results of figure 4 graphically: as presented, it is quite hard to determine how iPIP and cPIP affect PCNA and primase binding from the figure alone.

Point-to-point answer to the reviewer's comments

First of all, we would like to take the opportunity to thank the reviewers for their interest in our work and their useful comments on the manuscript. You will see we have taken their comments very seriously and have done our best to answer all of them.

REVIEWER 1

Madru and colleagues use an integrated approach that combines protein-protein interaction studies, X-ray crystallography and cryo-EM to describe the structure of the archaeal PolD-PCNA-DNA holoenzyme. They find that PolD contacts DNA via two distinct PIP motifs. Intriguingly, one of the PIP boxes is shared with the eukaryotic DNA polymerase Pol alpha. This is a very thorough study. In particular the cryo-EM structure reported (at resolution better than 3.8 Å) is a tremendous achievement. This study should definitely be published in Nature Communications, after minor revisions. I summaries below a few points that the authors should consider when preparing the revised version of their manuscript.

Major point:

1. Although the figures are very nicely prepared and clear, it would be extremely useful to include a movie separately depicting the structure of Pol D, the interaction between polymerase and DNA, polymerase and PCNA and PCNA and DNA.

We agree with the referee that a movie would be useful. A movie has thus been added to the supplementary data (S1 Movie). It accurately describes the defining features of the PolD-PCNA-DNA holoenzyme.

Minor points:

1. One key reference is incorrectly cited "March, M.D. et al." should be "De March, M. et al."

OK. Corrected

2. Related to the point above, a more thorough comparison should be drawn between the interaction mode observed between PCNA and DNA in the cryo-EM map of the archaeal holo-complex, and in the crystal structure of the human PCNA-DNA crystal structure. In particular, three distinct interaction modes between PCNA-DNA are resolved with focused classification and presented in Figure 2C. Are these interaction modes compatible with the cogwheel model proposed by De March et al?

With the structure of the PolD-PCNA-DNA complex, the structures of three structurally distinct classes of DNA polymerases have now been solved in complex with PCNA: the archaeal PolD-PCNA complex (a two-barrel fold DNAP) (this study), the bacterial PolIII-clamp-exonuclease-τ_c complex (a Polβ-like fold DNAP), and the eukaryotic Polδ-PCNA holoenzyme (a Klenow-like fold DNAP) (Lancey et al., 2019). In all three structures, the nascent DNA duplex runs straight through PCNA and adopts an almost perpendicular orientation with respect to the DNA. This view contrasts with the cogwheel mechanism proposed by De March and co-workers, where the PCNA adopts an eccentric and highly tilted position (~40°). However, this does not contradict the cogwheel sliding mechanism suggested

for PCNA when it is not associated with a DNAP. Indeed, DNA binding by the clamp is versatile and may be strongly influenced by the polymerase.

3. In the introduction the authors state "In eukaryotes, PCNA helps to recruit replicative DNAPs δ (Pol δ) and ε (Pol ε)". While this statement is correct for Pol δ, I am not sure that this is accurate for ε. In fact ε is recruited onto the CMG helicase before the initiation step of DNA replication. I suggest focusing on the observation that PCNA stimulates processive DNA synthesis of both leading and lagging strands.

We agree with the reviewer. This sentence has been revised as follows: "In eukaryotes, PCNA stimulates processive DNA synthesis of both lagging and leading strands upon association with DNAPs δ (Pol δ) and ε (Pol ε), respectively (Burgers and Kunkel, 2017; Chilkova et al., 2007; Nick McElhinny et al., 2008)."

4. The statement "The local resolution in the cryo-EM map surrounding the PCNA is about 4-4.5 Å" is unclear. I would say the outer perimeter of PCNA is 4-4.5 Å. Actually, would it not be better to claim 4-6 Å? (please note that my remark does not affect any of the conclusions of this study).

OK. This sentence has been modified as follows: "The local resolution of the outer perimeter of PCNA is 4.0-6.0 Å, substantially lower than the average resolution of the consensus map"

5. The sentence "The large proportion of polar contacts underlines the plasticity of the latter interaction, which contrasts with the site-specific PCNAclamp-1 interaction." is difficult to read and could be revised.

OK. This paragraph has been revised as follows: "This PCNA-clamp-2 interaction, which includes a large proportion of polar contacts, contrasts with the PCNA-iPIP site-specific interaction described above. The PCNA-clamp-2 interaction may thus be easily broken or profoundly remodelled, enabling the PCNA ring to form a new interface with PolD, when the polymerase encounters a damage and adopts an editing mode."

6. "suggesting that PolD is required for initiating DNA replication in Archaea". Archaea should be lower case.

OK. Corrected

7. Page 15. "The defocus range was between −0.5 and −3.5". This value does not match that report in Table S1.

OK. We checked the defocus range (see below). Aside from outliers, values are actually between -0.5µm and -3.5µm. The value in Table S1 have been modified accordingly.

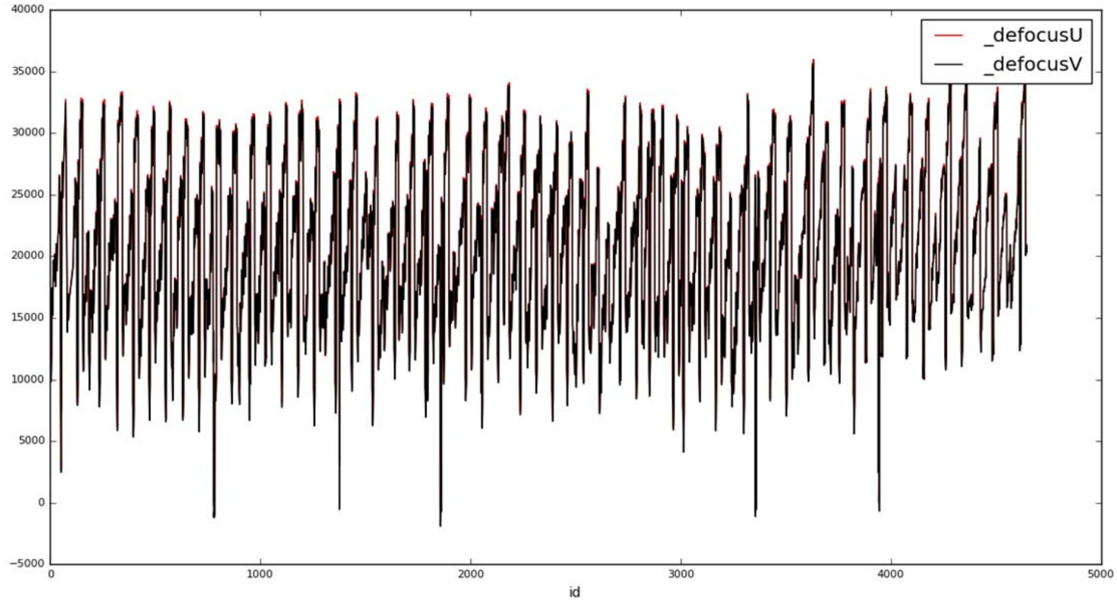

8. Figure S2 has a few problems.
Panel C, it would be good to overlay angular distribution and cryo-EM maps. OK. Done.
Panel D, arrow indicating rotation should be inverted!! OK. Corrected.
Panel E, should the threshold not be 0.143? OK. Corrected.

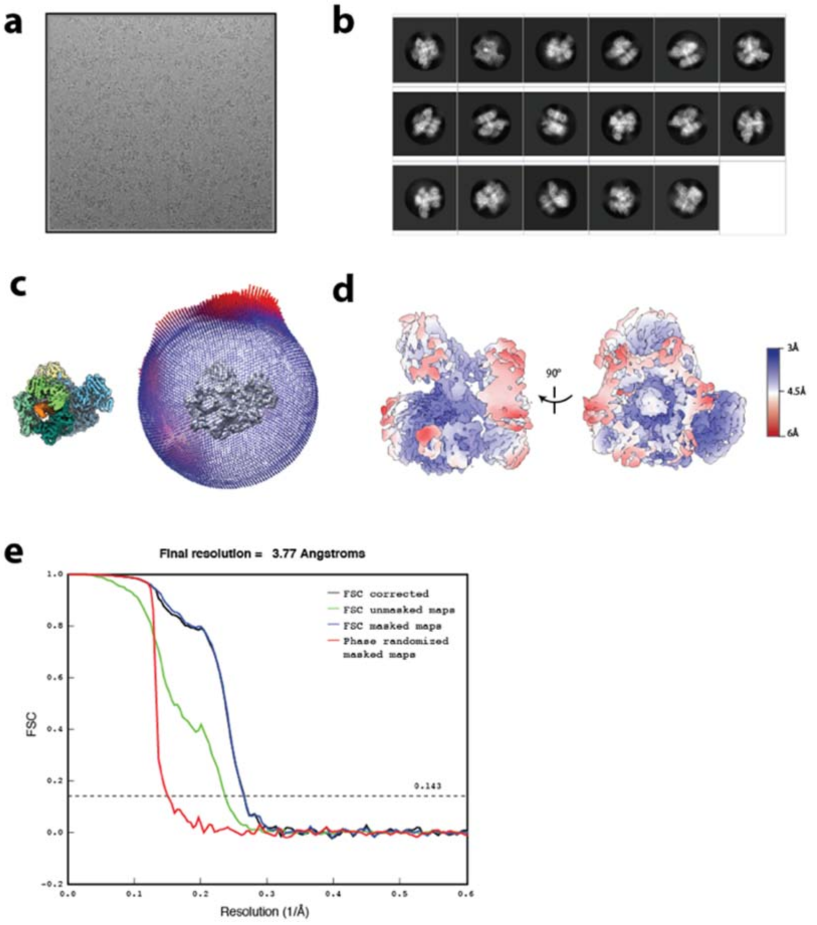

9. Table S1. Please revise "B -actors (Å2)". OK. Corrected.

Also consistency in measured defocus range (see methods, page 15). The same applies to map resolution range. Is it 3 to 4.5A as stated in ? 3 to 5A as stated in table S1? Or 3 to6 A as shown by the color code?

We carefully checked the local resolution map calculated by the *Locres* program in *Relion*. 3 to 6 is the correct range. Resolution values have been revised and harmonized throughout the manuscript.

REVIEWER 2

In this manuscript, Madru et al describe the cryo-EM structure of a PolD-PCNA-DNA complex from P. abyssi. In addition to reporting various aspects of the structure – many of which confirm previously reported interactions between individual components of the complex – the authors conduct a biochemical analysis of interactions made by the two PIP boxes on PolD. These results suggest that, while only one of the two PIP boxes can apparently interact with PCNA at a time, both are required for optimal PCNA binding. Additionally, the authors demonstrate that the C-terminal PIP (cPIP) is also capable of binding to primase via motifs similar to those found in eukaryotic polymerases.
Overall, I think that the combined structural and biochemical data provide nice insights into how archaeal PolD interacts with PCNA, and how this interaction may also allow primase to be transiently recruited. The study will be of interest to those working on the structure and evolution of DNA polymerases, and to the archael community. However, in light of a recent structure of human Pol ∂ in complex with PCNA and Fen1 (Lancey et al., https://doi.org/10.1101/872879), I am not convinced that this work will reach a broad audience. I have a couple of suggestions that I think would improve the manuscript, but ultimately think this manuscript is better suited to a more specialized journal.

Major critique:
I think that functional assays that extend beyond protein-protein interaction Kds would add a lot to this work. For example, how do cPIP and iPIP mutations affect priming, primer extension activity, and processivity of the PolD- PCNA complex on DNA at relatively physiological concentrations of protein and NTPs/dNTPs? Given the fairly high Kds of some of the interactions (e.g. 4 micromolar for the cPIP-Primase interaction), it would be interesting to determine whether these interactions are essential or more stimulatory.

The reviewer 2 is right, and we have performed activity assays (primer extension and processivity activity assays of the PolD-PCNA complex on DNA, including PolD wild-type and mutants where the cPIP or both iPIP and cPIP are deleted), which add a lot to this work. These new results are in perfect agreement with our structural data and our protein-protein interaction measurements.

These new results have been included to the revised manuscript:

"To assess the role of these two PIP-boxes, extension reactions were carried out in the presence of a primer-template of large size and increasing amounts of PCNA, to stimulate full-length DNA synthesis (**Figures 4A, 4B**). Using primed-M13mp18 DNA template, PolD progressively became stimulated upon increasing the concentration of PCNA. At 300 nM PCNA, the maximum amount of full-length DNA products was reached. However, full-length DNA synthesis by PolDΔiPIP-ΔcPIP, devoid of both C-terminal PIP-boxes, was never obtained upon increasing PCNA concentrations even at 300 nM PCNA. We verified that the abolishment of the functional interaction of PolDΔiPIP-ΔcPIP with PCNA was not due to an intrinsically catalytic-incompetent PolDΔiPIP-ΔcPIP, since full-length DNA products were detectable over a higher range of PolDΔiPIP-ΔcPIP mutant concentrations (**Figure S6**). In contrast, PolDΔcPIP, devoid of the C-terminal canonical PIP-box only, displayed functional interaction with PCNA, yielding similar amounts of full-length DNA products to PolD wild-

type (**Figures 4A, 4B**). Albeit the C-terminal canonical PIP-box (cPIP) is important for the physical interaction with PCNA (see below) (**Figures 4C, 4D**), these results show that the cPIP is dispensable for the processivity of the PolD-PCNA complex *in vitro*. Altogether, these results show that iPIP, but not cPIP, is required for the processivity of the PolD-PCNA complex. Consistently, while the iPIP was found to bind the PCNA PIP-binding pocket, the cPIP was not visible in the cryo-EM density of the PolD-PCNA-DNA ternary complex. Together these results suggest that PolD may be recruited by PCNA through a two-step mechanism (**Figure 4G**). First, PCNA is recruited by PolD through its interaction with the DP2 cPIP. Once the PolD-PCNA complex is loaded on DNA, the complex is stabilised by an interaction between PCNA and iPIP, as observed in the cryo-EM structure, while cPIP becomes dispensable."

These new results have been included to the main Figure 4 and to Figure S6:

Figure 4

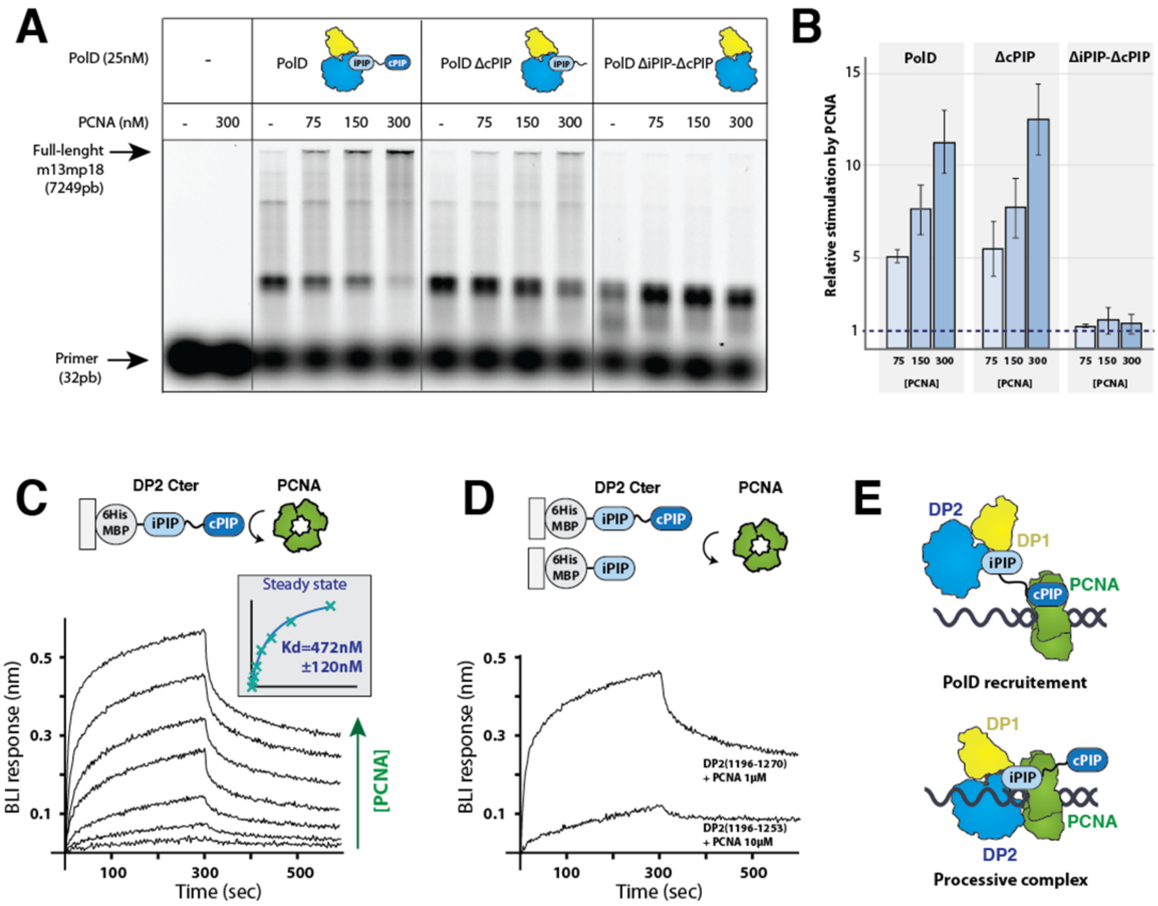

Figure S6

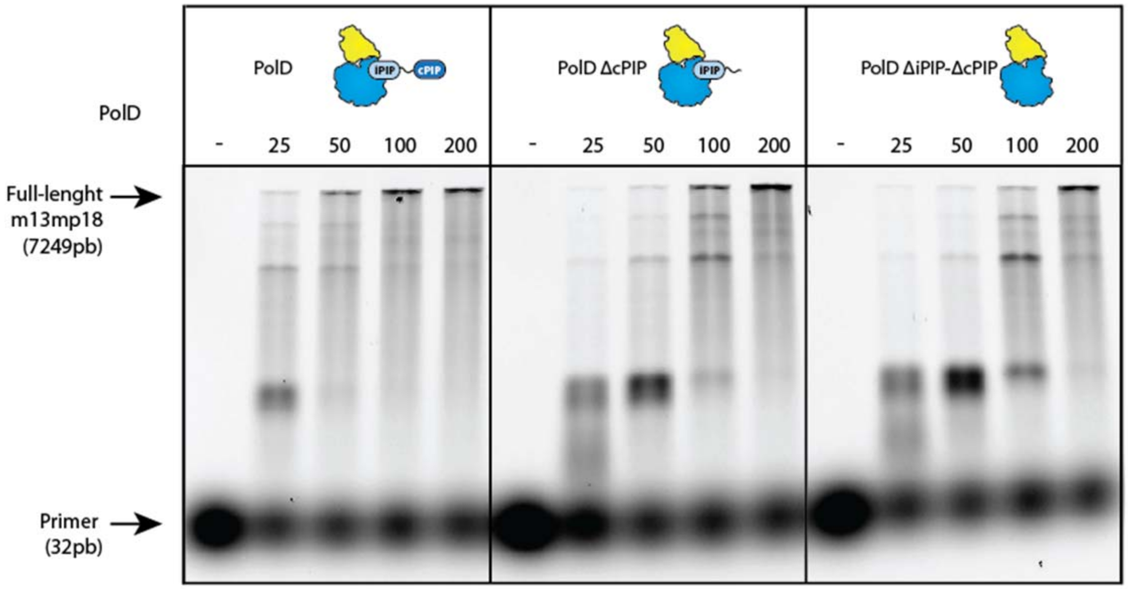

However, while studying how cPIP and iPIP mutations would affect priming is very interesting, it is a project on its own and it goes beyond the scope of this study.

Minor critique:
It would be helpful to summarize the results of figure 4 graphically: as presented, it is quite hard to determine how iPIP and cPIP affect PCNA and primase binding from the figure alone.

Figure 4, 5 and 6 have been profoundly remodeled to better summarize how iPIP and cPIP affect PCNA and Primase binding:

- Figures 4 now focuses on the interactions between PolD and PCNA, while Figure 5 focuses on the interactions between PolD and the primase. The role of both iPIP and cPIP are summarized by several diagrams (see below).
- Figure 6 has also been reworked. DNAPs representations have been lightened in order to highlight more clearly their structural similarities. In addition, PIP boxes are now colored according to their abilities to bind either PCNA, primase or both.

Figure 4:

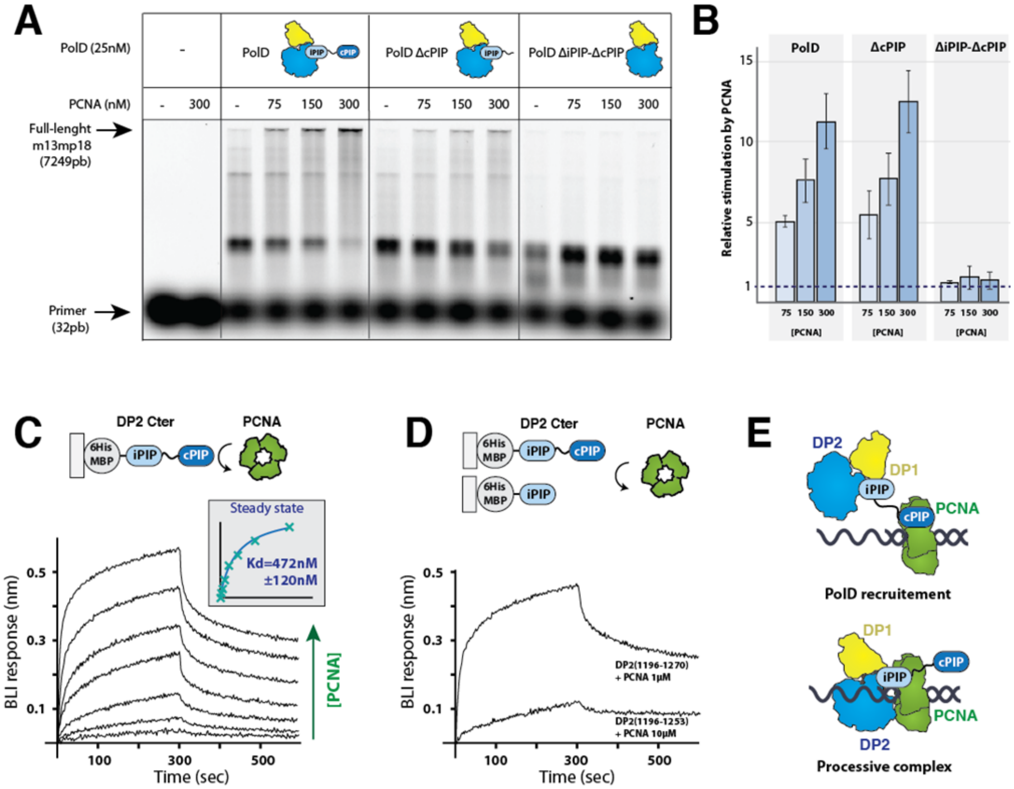

Figure 5:

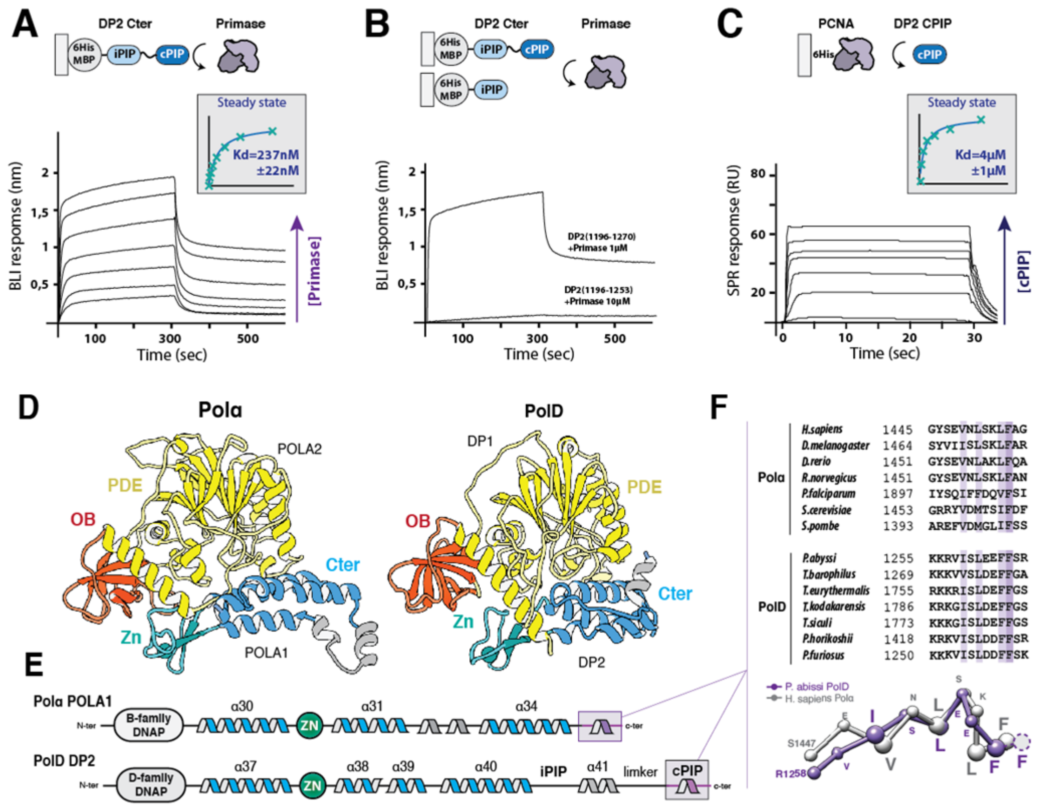
A
DP2 Cter
Primase
6His MBP
iPIP
cPIP
Steady state
Kd=237nM ±22nM
BLI response (nm)
Time (sec)
[Primase]
B
DP2 Cter
Primase
DP2(1196-1270) +Primase 1μM
DP2(1196-1253) +Primase 10μM
BLI response (nm)
Time (sec)
C
PCNA
DP2 CPIP
6His
cPIP
Steady state
Kd=4μM ±1μM
SPR response (RU)
Time (sec)
[cPIP]
D
Polα
PolD
POLA2
PDE
OB
Cter
Zn
POLA1
DP1
DP2
E
Polα POLA1
B-family DNAP
α30
α31
α34
ZN
PolD DP2
D-family DNAP
α37
α38
α39
α40
iPIP
α41
limker
cPIP
N-ter
c-ter
F
Polα
H.sapiens 1445 GYSEVNLSKLFAG
D.melanogaster 1464 SYVIISLSKLFAR
D.rerio 1451 GYSEVNLAKLFQA
R.norvegicus 1451 GYSEVNLSKLFAN
P.falciparum 1897 IYSQIFFDQVFSI
S.cerevisiae 1453 GRRYVDMTSIFDF
S.pombe 1393 AREFVDMGLIFSS
PolD
P.abyssi 1255 KKRVISLEEFFSR
T.barophilus 1269 KKKVVSLDEFFGA
T.eurythermalis 1755 RKKRISLDEFFGS
T.kodakarensis 1786 RKKGISLDEFFGS
T.siculi 1773 KKKGISLDEFFGS
P.horikoshii 1418 KRKVISLDDFFSR
P.furiosus 1250 KKKVISLDDFFSK
P. abissi PolD
H. sapiens Polα
S1447
R1258

Figure 6

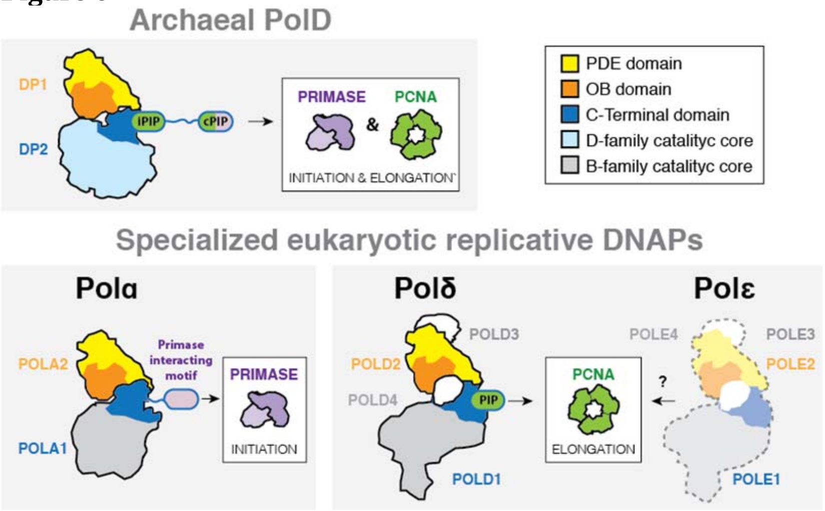
Archaeal PolD
DP1
DP2
iPIP
cPIP
PRIMASE
PCNA
&
INITIATION & ELONGATION
PDE domain
OB domain
C-Terminal domain
D-family catalityc core
B-family catalityc core
Specialized eukaryotic replicative DNAPs
Polα
POLA2
Primase interacting motif
PRIMASE
INITIATION
POLA1
Polδ
POLD3
POLD2
POLD4
PIP
POLD1
PCNA
ELONGATION
?
Polε
POLE4
POLE3
POLE2
POLE1

REVIEWERS' COMMENTS:

Reviewer #1 (Remarks to the Author):

The revised version of this manuscript is significantly improved.

The authors present key and novel structural and functional data that will be of great interest to researchers studying DNA polymerases and DNA replication in the three domains of life.

This is an excellent study and now deserves to be publish.

Reviewer #2 (Remarks to the Author):

The authors have satisfactorily addressed the comments in my initial review. The new functional assays are a very nice addition
